# Hierarchical Microstructure of Tooth Enameloid in Two Lamniform Shark Species, *Carcharias taurus* and *Isurus oxyrinchus*

**DOI:** 10.3390/nano11040969

**Published:** 2021-04-09

**Authors:** Jana Wilmers, Miranda Waldron, Swantje Bargmann

**Affiliations:** 1 Chair of Solid Mechanics, University of Wuppertal, 42119 Wuppertal, Germany; bargmann@uni-wuppertal.de; 2 Electron Microscope Unit, University of Cape Town, Cape Town 7701, South Africa; miranda.waldron@uct.ac.za; 3 Wuppertal Center for Smart Materials, University of Wuppertal, 42119 Wuppertal, Germany

**Keywords:** enameloid, microstructure, shark, teeth

## Abstract

Shark tooth enameloid is a hard tissue made up of nanoscale fluorapatite crystallites arranged in a unique hierarchical pattern. This microstructural design results in a macroscopic material that is stiff, strong, and tough, despite consisting almost completely of brittle mineral. In this contribution, we characterize and compare the enameloid microstructure of two modern lamniform sharks, *Isurus oxyrinchus* (shortfin mako shark) and *Carcharias taurus* (spotted ragged-tooth shark), based on scanning electron microscopy images. The hierarchical microstructure of shark enameloid is discussed in comparison with amniote enamel. Striking similarities in the microstructures of the two hard tissues are found. Identical structural motifs have developed on different levels of the hierarchy in response to similar biomechanical requirements in enameloid and enamel. Analyzing these structural patterns allows the identification of general microstructural design principles and their biomechanical function, thus paving the way for the design of bioinspired composite materials with superior properties such as high strength combined with high fracture resistance.

## 1. Introduction

**Figure 1:**
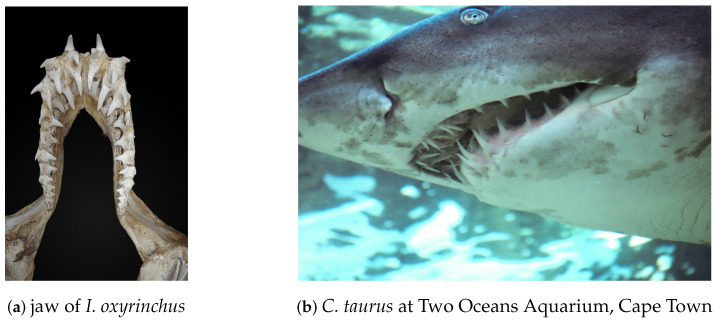
Shark teeth are arranged in series. Multiple rows of teeth grow behind each other and are continuously replaced over a lifetime. The primary function of the teeth of *I. oxyrinchus* (**a**) and *C. taurus* (**b**) is to pierce (not cut) prey. The jaws of both shark species can be opened very wide to swallow prey in one piece.

Shark teeth have always exerted a special kind of fascination on humans as they are the perfectly designed, highly efficient natural weapons of a deadly hunter. The teeth are arranged in multiple rows behind each other in the shark’s jaws (Figure 1) and exhibit a variety of shapes and sizes among different species, ranging from flattened domes over needles to triangular cutting tools with sharp, serrated edges. Even between closely related species, a wide variety of tooth shapes can be found [1] and may in fact be used to identify species [2]. These morphological variations have been attributed to differences in feeding behavior and, thus, mechanical loads [3,4,5]. Optimal functionality is further guaranteed by regular shedding and replacement of the teeth. The replacement rate varies between species and with age and water temperature. For spotted ragged-tooth sharks (*Carcharias taurus*), an average tooth loss rate of 1.06 teeth per day was identified [6], which means an individual spotted ragged-tooth shark will shed over 13,500 teeth in a lifetime. For other species, even more rapid replacement can be found [7,8].

Based on mechanical studies [3,9], it has been proposed that the frequent tooth replacement of shark teeth is due to wear rather than failure by tooth fracture. Wear on a small scale would not impact the structural strength of the tooth but would reduce its efficiency as a cutting or piercing tool. Studies aimed at understanding the biomechanics of shark teeth have found remarkably similar stress patterns even for drastically different tooth morphologies under static loading [9]. Dynamic tests show the increased cutting efficiency of serrated edges compared to smoother teeth, as well as their drastically faster mechanical wear [3]. In many shark species, however, tooth-on-prey contact leading to wear is comparatively rare as only large prey is manipulated with teeth [10].

It is well known that the mechanical properties of biological materials strongly depend on their complex microstructures. Hypermineralized tissues like shark tooth enameloid consist almost exclusively of nanoscale brittle mineral crystallites [4], yet exhibit considerable strength and exceptionally high fracture toughness way beyond that of the individual constituents. The relationship between the sophisticated microstructural hierarchy and the mechanical performance of other highly mineralized tissues such as amniote enamel or mollusc nacre has attracted considerable research interest, e.g., [11,12,13,14,15]. Shark enameloid, while similar in appearance and function to amniote enamel, is considerably less understood.

Enameloid consists of elongated fluorapatite (Ca_2_(PO_4_)F) crystallites with a roughly hexagonal cross-section visible in TEM images in different shark species [16,17]. The crystallites have a width of 50 nm to 80 nm and a length exceeding 1000 nm [17,18]. They are densely packed and arranged in a complex hierarchical structure. In all neoselachian sharks, the majority of the enameloid cover consists of bundled crystallite enameloid (BCE) and the tooth’s outer surface is covered in single crystal enameloid(SCE) (Single crystal enameloid is also commonly referred to as ‘shiny-layered enameloid’ reflecting its optical appearance) [19,20,21]. In the BCE, fluorapatite nanocrystallites are arranged in bundles that may be oriented longitudinally, radially, or circumferentially within the tooth, with slight variations occurring between different shark species [20,22]. In modern sharks (*selachimorpha*), bundles close to the dentine-enameloid junction are three-dimensionally interwoven, a structure type referred to as tangled bundled enameloid (TBE). Further from the dentine, the bundle arrangement becomes more regular, with the bundles aligned parallel to each other and to the tooth’s longitudinal axis, a structural motif referred to as parallel bundled enameloid (PBE).

This characteristic layered structure, is often referred to as a ‘triple layered structure’, with TBE, PBE and SCE each constituting one layer. However, the microstructure of shark enameloid has been found to be more complex than this description implies as it does not take into account, e.g., radial structural elements described in [21]. Instead, a nomenclature discerning the inner enameloid consisting of parallel bundled and tangled bundled enameloid, jointly referred to as the BCE unit, and an outer enameloid layer referred to as the ridge/cutting edge layer (RCEL) has been suggested in [19]. The RCEL itself consists of an external layer of single crystal enameloid and an internal layer of circumferential bundles. The inner and the outer enameloid are separated clearly as visible in micrographs, while transitions between the substructures within each layer are generally smooth. Elemental analysis of shark tooth enameloid shows that the crystallite composition varies slightly between the inner and the outer enameloid, with the outer enameloid being richer in the substituting ions magnesium and sodium than the inner, bundled enameloid [18].

The enameloid microstructures in other neoselachian species vary more drastically from the patterns identified in modern sharks. Batoids (rays and skates), for instance, exhibit a less complex microstructure, with some species even losing bundles completely [23,24].

This study characterizes and compares the enameloid microstructure of two modern lamniform sharks, *Isurus oxyrinchus* (shortfin mako shark) and *Carcharias taurus* (spotted ragged-tooth shark). A description of enameloid hierarchical microstructure in the style of the established description of amniote enamel [14,25,26] is proposed. As amniote enamel serves the same protective function as enameloid, structural similarities may be indicative of biomechanical function. Such similarities between enameloid structure and the enamel of different mammalian species are identified and discussed here. By focusing on structural motifs and patterns in shark enameloid, the foundation for microstructure design in synthetic composite materials is laid. As biological materials exhibit sophisticated structures on multiple length scales, they commonly combine highly desirable properties such as high fracture toughness and strength which are difficult to obtain in typical engineering materials [27]. Thus, mimicking some of nature’s microstructural design principles may be the key in designing high performance composites.

## 2. Materials and Methods

Teeth of two species of the order *Lamniformes*, the spotted ragged-tooth or sand tiger shark (*Carcharias taurus*) and the shortfin mako shark (*Isurus oxyrinchus*), were studied. Both species feed mainly on bony fish and occasionally other elasmobranches or even sea turtles. Their teeth are used primarily to incapacitate prey before ingestion or, less often, to rip pieces from larger prey.

**Figure 2:**
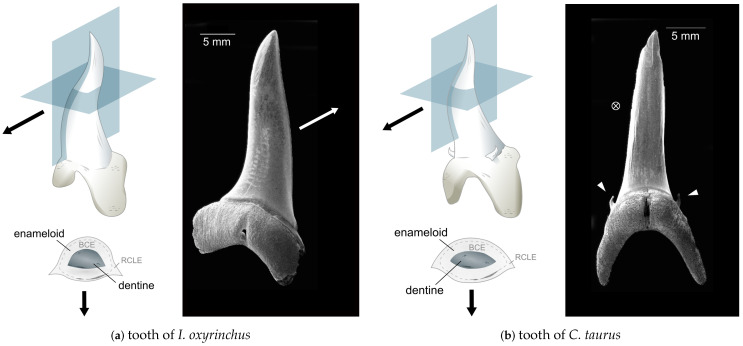
Tooth morphology of the two studied species. Both species have slender, dagger-like teeth without serrated edges. The teeth are curved slightly to improve the grip on prey. Sketches on the left show the tooth and its transversal cross-section. Arrows indicate the facial direction. The shaded planes correspond to the section planes used in imaging. The enameloid cover consists of inner and outer enameloid, marked in the cross-section with BCE and RCLE, respectively. *I. oxyrinchus* teeth (**a**) are monocuspid while *C. taurus* teeth (**b**) are tricuspid with a large main cusp and small outer cusplets indicated by white arrowheads. The *I. oxyrinchus* tooth (**a**) exhibits a small amount of wear on the lingual side of the tip. The *C. taurus* tooth depicted in (**b**) is fractured at the tip.

Samples of *I. oxyrinchus* teeth were acquired from Hout Bay, South Africa. The *C. taurus* teeth samples had been shed naturally and were kindly provided by Two Oceans Aquarium, Cape Town, South Africa. The teeth originate from one or more of the 5 adult females living in captivity (but born free) at the aquarium, weighing 80 kg to 170 kg. Teeth samples were stored at ambient conditions before specimen preparation and imaging. Teeth of both species were placed in a mold, covered in Spurrs resin and put in an oven at 60 °C for 12 h to allow the resin to set. The teeth were then ground in the longitudinal and transverse sections (Figure 2) and the exposed areas were polished with 0.2 μm aluminum powder. The samples were sonicated at 50 Hz in distilled water for 2 min and then etched with 10% hydrochloric acid for 2 min and rinsed in distilled water for 5 min. They were carbon coated in an evaporation coater and viewed with a Tescan MIRA SEM at 5 kV. The SEM images for both species at different magnifications are gathered in the Supplementary Material Figures S1–S15.

## 3. Results: Microstructure Description of Enameloid in *I. oxyrinchus* and *C. taurus*

The teeth of both studied species, *I. oxyrinchus* and *C. taurus*, have remarkably similar morphologies (Figure 2). The teeth are slender and needle-like and possess no serrated edges. This morphology is commonly associated with piercing of prey [5]. Both are curved in an S-shape with the tooth protruding forward and then curving backward into the oral cavity and only the tip curving forward again. The anterior side especially of *I. oxyrinchus* teeth is flattened. The most pronounced difference between teeth of the two species is the presence of small tricuspids in *C. taurus* not found in *I. oxyrinchus*.

The teeth imaged in this work were approximately 10 mm in length and had a lenticular cross-section with a width of 2.5 mm in the upper region where the teeth were sectioned. Other samples were larger, with lengths reaching 15 mm for *I. oxyrinchus* and 20 mm for *C. taurus*. The enameloid cover in the studied transversal sections had a thickness of ca. 0.5 mm for *I. oxyrinchus* and ca. 0.4 mm for *C. taurus*. The enameloid thickness varied slightly between different teeth; in the longitudinally sectioned tooth of *C. taurus*, the thickness on the anterior side of the tooth was 0.4 mm and on the posterior side it was 0.7 mm.

### 3.1. Enameloid Microstructure of Isurus oxyrinchus

**Figure 3:**
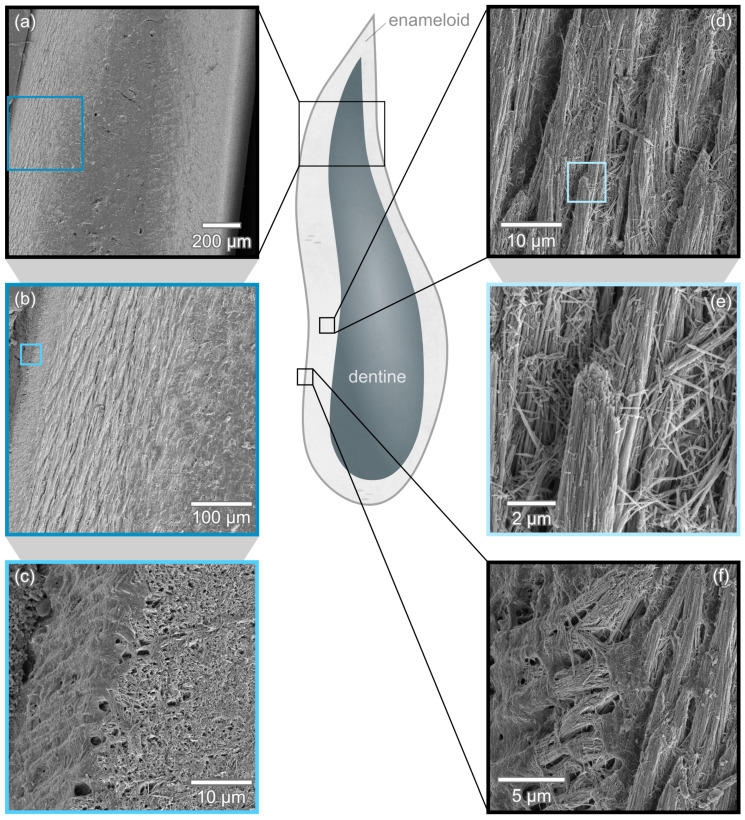
*I. oxyrinchus* tooth in longitudinal section. The enameloid cover is characteristically layered as seen in (**a**,**b**). Zooming into the outer enameloid, a densely packed outer layer is found in the top part of the tooth (**c**) while in the lower part, distinct circumferential bundles are apparent (**f**). (**d**) The outer enameloid surrounds a layer of parallel aligned crystal bundles. (**e**) Magnified view of the broken tip of a bundle from (**d**).

The enameloid cover of *I. oxyrinchus* teeth exhibits the typical layered structure in longitudinal section as seen in Figure 3a,b. The outermost layer with a thickness of 30 μm to 35 μm consists of densely packed crystals (Figure 3c) in the upper region of the tooth. In the lower region, no single crystal enameloid is visible and the outer enameloid consists solely of a single layer of circumferentially oriented bundles as seen in Figure 3f.

**Figure 4:**
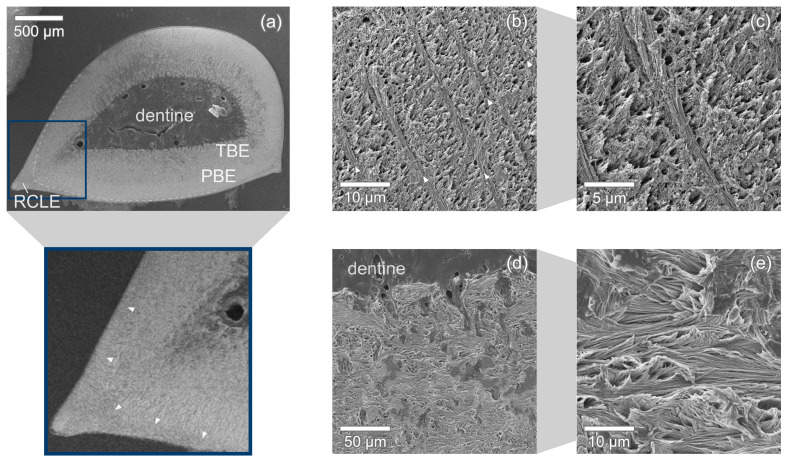
Transversal section of *I. oxyrinchus* tooth. The tooth’s enameloid cover is built up in layers (**a**). The outer enameloid is designated RCLE and clearly separated (dashed line, arrow heads in zoomed-in view) from the inner enameloid consisting of PBE and TBE. (**b**,**c**) show the outer edge of the enameloid cover and radial elements (arrow heads) running towards the outer enameloid. In between the radial elements, bundles are oriented parallel to the tooth’s longitudinal axis. (**d**,**e**) show three-dimensional entanglement of bundles close to the dentine-enameloid junction.

The outer enamel covers a layer of parallel bundled enameloid (Figure 3d,e) with a thickness of ca. 170 μm. In this region, crystal bundles are aligned parallel to each other and oriented along the tooth’s longitudinal axis. Closer to the dentine, this clear arrangement becomes less regular and transitions smoothly into entangled bundles. In the longitudinal section depicted in Figure 3, the transition from dentine in the tooth’s core to the enameloid cover appears smooth (Figure 3b) due to only minor etching. In the transversal section Figure 4a, a sharp junction between enameloid and dentine is apparent.

**Figure 5:**
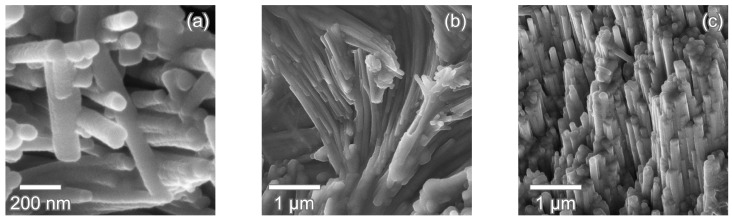
Fluorapatite crystallites in *I. oxyrinchus* tooth. (**a**) Fracture surface showing individual crystallites. (**b**) The thin, rod-like crystallites change orientation in the TBE (transversal section). Neighboring crystal rods remain parallel. (**c**) Densely packed crystallites within a crystallite bundle (transversal section). The crystallites are aligned parallel to each other and in close contact with each other.

The bundles in the inner enameloid have a diameter of ca. 4 μm to 9 μm and consist of densely packed crystallites with an average width of 66 nm visible in Figure 5a. This size is in good agreement with the range of 50 nm to 80 nm found for *I. oxyrinchus* in [17,18]. The crystallites are several micrometers in length, but an exact measurement is impossible in the micrographs as the visible length exceeds the field of view. Within a bundle, the elongated crystallites are aligned parallel to each other without visible pores or gaps between them (Figure 5c). Neighboring crystallites remain in contact in the TBE when the bundle curves and changes direction (Figure 5b).

Inner, bundled enameloid and outer enameloid are clearly separated with a distinct boundary clearly visible in the medial and distal tooth edges in transversal section Figure 3a. In these edges, the outer enameloid is thicker while in the middle of the lingual and labial faces, the outer enameloid’s thickness is 30 μm to 35 μm as found in the longitudinal section.

In the transversal section, the different regions of the inner enameloid are more distinct due to stronger etching. Adjacent to the enameloid-dentine junction, crystallite bundles are three-dimensionally interwoven (Figure 4d,e). The TBE is approximately 270 μm to 290 μm thick. The surrounding PBE region is only marginally thinner. The inner enameloid, thus, appears to be made up of equal amounts of TBE and PBE. Exact measurements, however, are difficult as the transition between the regions is smooth.

The parallel bundles of the inner enameloid are interspersed with regularly arranged radial elements with a thickness of ca. 1.5 μm (Figure 4b,c). The distance between two radial elements ranges from 6 μm to 10 μm. This corresponds to the diameter of an individual bundle, thus, the radial elements appear to separate individual rows of parallel aligned bundles. The crystallites within the radial elements are oriented at almost a $90^{\circ }$ angle to the bundle direction (i.e., parallel to the imaging plane) and run from the enameloid-dentine junction radially outwards to the outer tooth surface. As shown in Figure 4b, the radial elements merge into the outer enameloid layer which consists of densely packed, randomly oriented crystallites.

### 3.2. Enameloid Microstructure of Carcharias taurus

**Figure 6:**
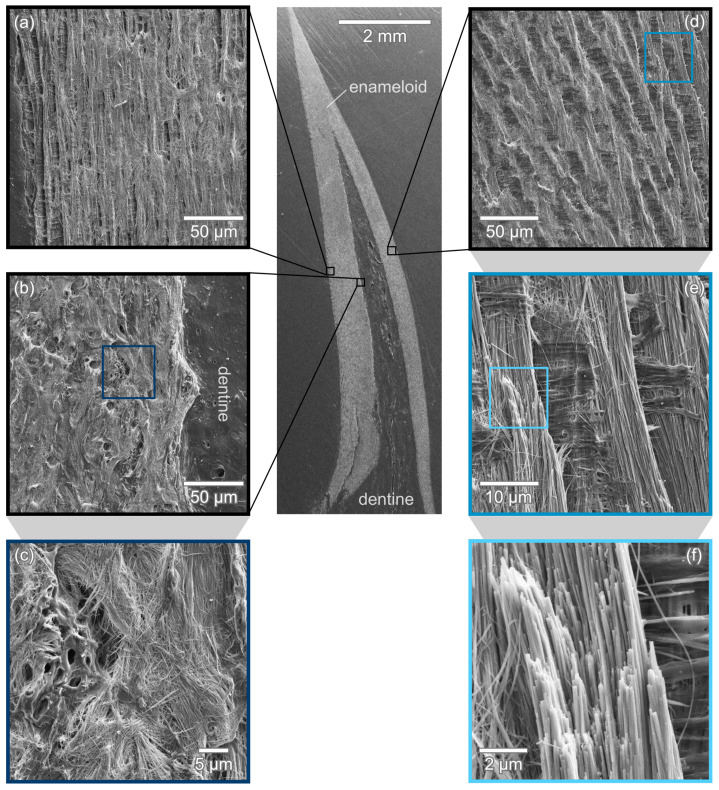
*C. taurus* tooth in longitudinal section. In the area imaged in (**a**), parallel bundle enameloid (PBE) reaches all the way to the outer surface which is obscured by embedding material. (**b**,**c**) show three-dimensionally entangled bundles belonging to the TBE close to the dentine. (**d**–**f**) show a sequence of images with increasing magnification into the PBE in the outer range of the inner enameloid. Between the bundles, remnants of layers between the bundles are visible. An individual bundle (**f**) consists of densely packed, parallel aligned crystallites.

Figure 6 depicts the longitudinal section of a *C. taurus* tooth. In this section, no outer enameloid is visible and the PBE appears to extend right to the tooth edge (Figure 6a,d) with no outer enameloid visible. A thin layer of outer enameloid or remainders of one could possibly be obscured by the embedding material covering the tooth’s edge. The PBE transitions smoothly into a region of TBE with three-dimensionally interwoven crystallite bundles as visible in Figure 6b,c. The enemaloid-dentine junction is sharp and clearly visible with no dentine reaching into the enameloid.

**Figure 7:**
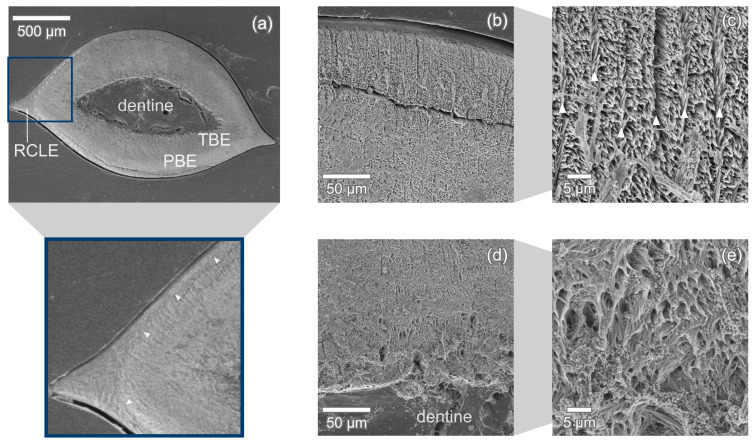
Transversal section of *C. taurus* tooth. (**a**) The enameloid cover is made up of an outer enameloid (RCLE) clearly separated (dashed line, arrow heads in zoomed-in view) from the inner enameloid which itself consists of TBE and PBE. (**b**,**c**) In the middle of the tooth edge, the outer enameloid appears to be worn away and parallel aligned crystal bundles (PBE) intersected by evenly spaced thin radial elements (arrow heads) are visible. (**d**,**e**) In the inner enameloid close to the dentine, the bundles are interwoven and change orientation frequently in all directions. The crack apparent in (**a**,**b**) likely occurred during specimen preparation.

In the transversal section in Figure 7a, the typical layered structure of modern shark’s enameloid is readily identifiable. The outer enameloid is clearly visible in the medial and distal tooth edges and narrows drastically towards the middle of the lingual and the labial faces. In the middle of these tooth faces, the outer enameloid appears to vanish completely (Figure 7b).

The transition from parallel aligned crystallite bundles (PBE) to the entangled, interwoven bundles (TBE) in the inner enameloid is smooth without any sharp boundaries. The PBE region has a thickness of about 115 μm measured in the transversal section Figure 7. The TBE with a thickness of ca. 215 μm is significantly thicker.

**Figure 8:**
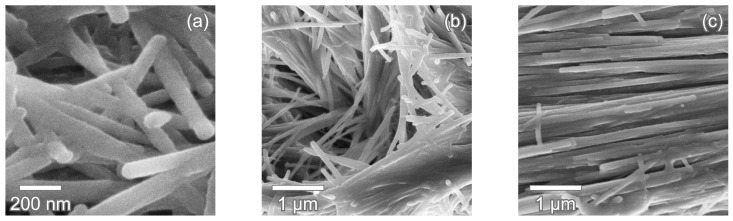
Fluorapatite crystallites in *C. taurus* tooth. (**a**) Individual crystallites at the polished sectioning plane. (**b**) Crystallites curve and change orientation in the TBE while remaining parallel to their neighbors (transversal section). (**c**) Within a bundle in the inner enameloid, crystallites are in close contact with each other and aligned along their long axis (transversal section).

The crystal bundles in the inner enameloid have a diameter of 5 μm with the crystals’ long axes running parallel to the bundle’s long axis, as seen in Figure 6e,f. Figure 8 shows close-up micrographs of fluorapatite crystallites. The crystallites have an average thickness of 60 nm and are aligned along their elongated longitudinal axes. The crystallites are densely packed within a bundle and appear to be in direct contact along the whole crystallite length. This contact and the alignment are maintained even when the crystallites curve and change direction in the TBE (Figure 8b).

**Figure 9:**
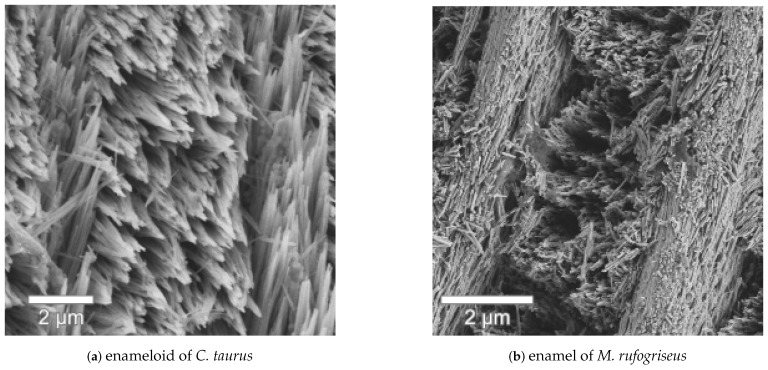
Transversal sections of (**a**) the outer enameloid of *C. taurus* and (**b**) the modified radial enamel in a tooth of *Macropus rufogriseus* (red-necked wallaby). Both species exhibit radial sheets of densely packed crystallites separating rows of bundled crystallites. The hydroxyapatite crystallites of the mammalian enamel are thinner than the fluorapatite of enameloid but the higher-level bundles and radial elements have the same size in both tissues. Despite the significant size difference between the two species, the imaged teeth are of similar size with a crown height of 10 mm in *C. taurus* and ca. 7 mm in *M. rufogriseus*.

Close to the edge, radial elements intersecting the transversal plane at approximately $90^{\circ }$ can be seen in Figure 7b,c. Each of these radial elements has a thickness of ca. 1.5 μm and the distance between two elements is approximately 5 μm or slightly higher (up to 7.5 μm) which correlates to one bundle diameter. The crystallites within the radial elements are aligned in parallel with each other and their long axes are oriented at almost a $90^{\circ }$ angle to the long axes of the bundles as seen in Figure 9a which shows two radial elements with the crystallite bundles between them.

## 4. Microstructural Features of Modern Shark Enameloid

### 4.1. Lamniform Shark Enameloid Structure

*Isurus oxyrinchus* and *Carcharias taurus* exhibit a remarkably similar enameloid microstructure corresponding to the typical layered structure known for modern sharks [19,20]. The enameloid of non-selachian species shows drastically varying levels of complexity with some exhibiting crystallite bundles [24] while the fossilized teeth of some *Ctenacanthiformes* are so highly mineralized that individual crystallites cannot be distinguished [28].

The microstructural complexity in modern sharks has been suggested to be a functional adaptation to the specialized feeding behavior in comparison to other chondrichthyes [28]. The enameloid microstructure of *I. oxyrinchus* and *C. taurus* enameloid described here exhibits a clear hierarchical set-up. It consists of thin, needle-like crystallites with a length exceeding a few micrometers. The crystallites of both species have an average width of ca. 60 nm to 65 nm, which falls perfectly into the range found in [18] for *I. oxyrinchus* and is in the same range as the 50 nm identified in [17]. Table 1 summarizes the sizes of all structural features in both species.

The majority of the enameloid cover is composed of these crystallites, arranged in densely packed crystallite bundles. The enameloid region adjacent to the dentine is in both species characterized by intertwining bundles, corresponding to the TBE. In *C. taurus*, the boundary between dentine and TBE is well-defined while in *I. oxyrinchus*, especially in longitudinal section (Figure 3), the transition is smooth and some dentine extends into the enameloid cover. A similarly smooth transition has been found for *I. oxyrinchus* in [4] and for *C. taurus* in [29].

The TBE is covered by PBE in which the crystallite bundles are aligned parallel to each other and the tooth’s longitudinal axis in both studied species. In *I. oxyrinchus*, TBE and PBE regions are of similar thickness while in *C. taurus*, the TBE layer makes up approximately two thirds of the bundled enameloid unit (Table 1).

The outermost layer of a modern shark’s tooth typically consists of single crystal enameloid in which crystals are oriented randomly. In the studied teeth of *I. oxyrinchus*, this outer layer is visible and in the lower regions of the tooth, a single layer of circumferential bundles as also found in [18] can be identified. The outer enameloid in *I. oxyrinchus* here is with ca. 30 μm thickness significantly thicker than the one described in [18] for the same species.

For *C. taurus*, no outer enameloid layer could be found in longitudinal section. The outer enameloid is thick in the tooth’s medial and distal edges and thins over the tooth’s circumference until it vanishes in the middle of the lingual and labial faces (Figure 7). The *C. taurus* tooth studied in [29] exhibits an outer enameloid layer of approximately uniform thickness. This suggests that a thin outer layer might have been worn away, either during normal function, after shedding due to environmental conditions [10] or during etching. The outer enameloid of *C. taurus* does not exhibit circumferential bundles as are found in *I. oxyrinchus*. This is the most significant difference in the enameloid microstructures of the two species.

From the outer enameloid, thin radial elements reach into the inner enameloid where they intersperse individual rows of parallel bundles. The presence of such radial elements has been documented for *I. oxyrinchus* [18], *C. taurus* [29] and numerous other lamniform shark species [19,20,33].

### 4.2. Radial Elements in Enameloid

Generally, the radial elements found in the outer enameloid and PBE are referred to as ‘radial bundles’ [19,20]. Their geometry is described as ‘ribbon-like’ bundles in *I. oxyrinchus* [18]. FIB-SEM tomography of the enameloid of two carcharhiniform sharks [33] shows the three-dimensional arrangement of radial elements close to the SCE. The crystallites are arranged within a thin layer between the bundles with frequent gaps in the layer and transversal connections between adjacent layers apparent.

In the studied section of *I. oxyrinchus*, the crystals within the radial elements are aligned parallel to the transversal section (Figure 4) while in the transversal section of *C. taurus* (Figure 7), the crystallites in the radial elements intersect the imaging plane at an angle, compare also Figure 9a. From these micrographs, the radial element layers appear to be continuous thin sheets with two directions (the radial and the axial one) being much larger than the thickness and have no gaps readily apparent. The sheets separate rows of crystal bundles in which the crystals are oriented at an angle to the sheet crystals. In both species, this angle appears to be close to $90^{\circ }$.

This structural motif of thin sheets separating rows of crystal bundles from each other can also be found in the dental enamel of some herbivorous mammals [34,35] and is there referred to as *modified radial enamel*. Figure 9 shows the radial elements in *C. taurus* enameloid and the modified radial enamel in a molar of *Macropus rufogriseus*, the red-necked wallaby, showing the striking similarity between the structures. The crystallites in the marsupial’s enamel are hydroxyapatite and slightly thinner than the fluorapatite crystals of *C. taurus*’s enameloid. Similarly, the bundles are thinner than in the enameloid, but the sheets have the same thickness of 1.5 μm (Table 1). The similar length scale of both structures is remarkable when considering the large size difference between the two species. The investigated teeth are of similar size with a crown height of 10 mm in *C. taurus* and ca. 7 mm in *M. rufogriseus*. The diameter of the shark tooth is with 2.5 mm of the same order as an individual cusp of *M. rufogriseus*’s molar. For mammalian enamel, the size of microstructural elements is independent of the size of a single tooth or the animal [14].

Modified radial enamel in mammals is generally interpreted as an adaptation to large axial stresses arising due to horizontal mastication movements that are especially common in grazing species. These axial stresses favor crack propagation in radial direction, i.e., cracks travelling perpendicular to the tooth’s longitudinal axis from the surface towards the softer dentine. Modified radial enamel introduces vertical decussation planes, i.e., planes of abrupt crystal orientation discontinuities, within the crack path. This results in deflection of the crack and twisting of the crack surface, thus, effectively slowing or arresting cracks before reaching the dentine and protecting the tooth from catastrophic fracture [14]. Slender shark teeth like the ones studied here are loaded in bending during holding and shaking of prey [9]. This loading case results in dominant axial tensile stresses which would cause radial crack growth. The decussation planes introduced by the presence of radial sheets, thus, can distort the crack path. The crystallite bundles are oriented axially and, thus, are stiff under bending and do not exhibit significant weak propagation paths for radial cracks. The radial elements, therefore, provide toughening against chipping of the tooth, i.e., crack propagation parallel to the tooth surface, without introducing additional weakness against crack propagation under bending.

## 5. Structural Hierarchy in Dental Materials: Similarities between Enameloid and Enamel

**Figure 10:**
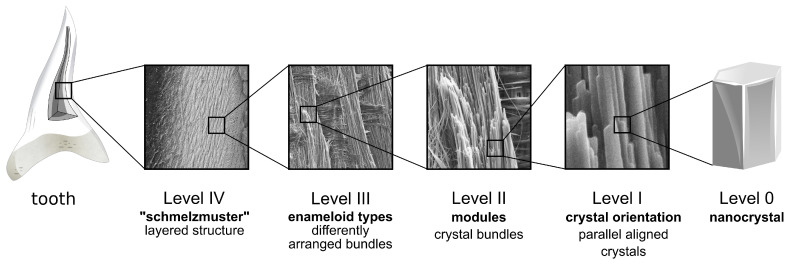
Structural hierarchy of shark teeth enameloid. The enameloid cover of an individual shark tooth exhibits a five-level hierarchical microstructure. On Level IV, the *schmelzmuster*, different structural patterns are combined to improve the biomechanical function of the tooth. These Level III patterns are the different enameloid types and consist of the Level II enameloid modules. In shark enameloid, the most common module is a bundle of parallel aligned crystallites (Level I). The lowest level of the hierarchy, Level 0, is the individual nanoscale crystallite.

Shark enameloid and amniote enamel consist of different minerals and have been shown to differ in evolutionary origin [19,36] but both dental tissues serve the same function as the outermost layer of the tooth that protects the softer dentine. Thus, identification of common microstructure patterns—such as radial elements—can give valuable insight into biomechanical function, as seen in Section 4.2, and may form the basis for the design of bioinspired composite materials. Therefore, the hierarchical microstructure of shark enameloid is in the following described in comparison with amniote enamel for which a well-established terminology exists ([26], see also [14]). Remarkably, both tissues exhibit the same five levels of hierarchy, depicted in Figure 10.

The smallest building block in each tissue is the individual nanocrystal which is designated as Level 0 of the hierarchy. Level I describes the local arrangement of crystallites. In the shark enameloid studied here, individual crystallites are either aligned in parallel or—in the shiny layer enameloid—fully randomly arranged. The parallel aligned enameloid crystals are densely packed into crystallite bundles on Level II which in the enamel nomenclature is also referred to as the *module* level. In dental enamel, different modules can be identified [14] but the most prominent are the ‘rods’ or ‘prisms’ typical for mammalian enamel. These generally have a rounded cross-section with diameters of 2 μm to 10 μm and, within the rods, enamel crystallites are predominantly arranged parallel to each other and to the rod’s length axis. This arrangement is identical to the bundles in shark enameloid.

The assembly of these modules forms Level III of the hierarchy. Different assemblies such as TBE and PBE can be referred to as enameloid types, in analogy to ‘enamel types’. Much of the functional adaptation of mammalian enamel occurs on this third level of the hierarchy that is characterized by locally varying arrangements of modules and single crystallites as is the case in the discussed parallel bundled enameloid interspersed with radial elements. Over the thickness of the enameloid cap, different enameloid types occur, and some enameloid types may only be found in certain regions of the tooth. For *I. oxyrinchus* and *C. taurus*, the enameloid consists of an innermost layer of TBE, surrounded by PBE which in turn is covered by the outer enameloid. This varying pattern constitutes Level IV of the structural hierarchy and is the equivalent to the *schmelzmuster* (from the German ‘Zahnschmelz’ for tooth enamel) of enamel.

In many evolved mammalian species, the enamel consists of an inner layer of decussated enamel types in which rods are interwoven or arranged at sharp angles to each other [14]. This inner layer is commonly covered by radial enamel in which the rods aligned parallel and the whole tooth is covered in a thin layer of “prism-less”, i.e., single crystallite enamel. This typical schmelzmuster is interpreted to be an adaptation for fracture resistance, as cracks can easily travel along the boundaries between parallel aligned bundles but the presence of decussation deflects and bifurcates the crack path, resulting in toughening and arrest of the crack before critical failure. The layered structure of a shark tooth is a clear match for this set-up, suggesting that the parallel bundled enameloid increases stiffness of the tooth necessary for piercing while the tangled bundled enameloid increases fracture resistance. Strikingly, the relative thickness of the TBE layer found in *I. oxyrinchus* and *C. taurus* in this study (Table 1) corresponds exactly to the 50% to 65% of decussated enamel found in mammalian enamel [14].

Remark: The structural hierarchy of *Isurus oxyrinchus* teeth is also discussed in [18]. A six-level hierarchy description is proposed in which the fluorapatite unit cell constitutes Level 1 and, thus, the smallest building block and Level 6 describes the whole tooth. The higher-level structural features identified in [18] correspond to Levels II–IV described above.

## 6. Conclusions

Hard tissues such as shark enameloid are biological composites of nanoscale mineral crystals arranged in intricate hierarchical patterns interspersed with only minor amounts of remnant protein. The microstructural design in these tissues results in a macroscopic material that is stiff, strong and tough despite consisting almost completely of brittle mineral.

Analysis of micrographs of two lamniform shark species, the shortfin mako (*Isurus oxyrinchus*) and the spotted ragged-tooth shark (*Carcharias taurus*), reveals the hierarchical structure of enameloid. Fluorapatite nanocrystallites (Level 0) are arranged in bundles (Level II) that themselves are arranged in a layered pattern over the enameloid cover (Level IV). The microstructural arrangements found in both species are remarkably similar, both contain parallel aligned bundles (PBE) interspersed with radial elements and an inner layer of three-dimensionally interwoven TBE. In this work, the hierarchy of enameloid microstructure is discussed in comparison to the well-established nomenclature used to describe the microstructure of amniote enamel [26]. The microstructures of both tissues exhibit the same five hierarchical levels, despite their different evolutionary origin.

Remarkably, on different hierarchical levels, direct structural analogues for shark enameloid microstructure patterns could be identified in mammalian enamel. Shark enameloid contains radial elements separating rows of crystal bundles on Level III, a structural motif strikingly similar to the mammalian *modified radial enamel*. Another Level III enameloid type, the tangled bundle enameloid, is structurally identical to the decussated *irregular enamel* of marsupials and proboscideans [35,37]. On Level IV, these different enameloid types are arranged in a layered structure that corresponds to the typical schmelzmusters of mammalian enamel.

These striking structural similarities allow one to draw conclusions on enameloid microstructure function based on the wealth of information available for enamel [14]. Modified radial enamel, for instance, has been found to increase fracture resistance while maintaining high stiffness. Likewise, the radial elements in enameloid are likely to increase fracture toughness by providing ‘easy’ crack propagation paths along the discontinuities in crystallite orientation. These deliberately introduced crack paths result in energy dissipation through crack deflection and can guide fracture away from sensitive parts of the tooth. Similarly, the characteristic layered structure of lamniform shark enameloid is functionally identical to mammalian enamel with an inner decussated enamel providing fracture toughness and protection of the soft dentine from cracks.

By transferring such biological design principles to synthetic materials, unique property combinations such as high strength combined with high fracture resistance may be achieved. Mimicking the full hierarchical structure of a biological tissue over all involved length scales may still be unachievable with modern fabrication techniques, but adopting individual structural motifs has been used successfully in the development of ‘self-sharpening’ knives inspired by rat teeth [38] or impact resistant glass based on nacre [39]. As the described structural motifs of radial elements and the layered structure have developed independently in the different tissues, they should be investigated further with regards to the toughening mechanisms they provide and to derive design principles to be applied in bioinspired composites and metamaterials.

## Figures and Tables

**Table 1:** Size of structural features in modern shark enameloid and mammalian enamel.

	*I. oxyrinchus* Enameloid	*C. taurus* Enameloid	Wallaby Enamel	Human Enamel
thickness TBE [μm]	270–290	215	-	-
thickness PBE [μm]	250	115	-	-
thickness outer enameloid [μm]	30–35	10–30	-	-
crystal width [nm]	50–75	55–70	45–50	50–70 [30,31]
bundle diameter [μm]	4–9	5	3–4	4–8 [32]
thickness of radial elements [μm]	1.5	1.5	1–1.5	-
distance radial elements [μm]	6–10	5	4	-

## Supplementary Materials

The SEM micrographs of the studied teeth are available online at https://www.mdpi.com/article/10.3390/nano11040969/s1, Figures S1–S15.
