# Supplementary material for: Hierarchical Microstructure of Tooth Enameloid in Two Lamniform Shark Species, Carcharias taurus and Isurus oxyrinchus
Source: Nanomaterials (Basel). 2021 Apr 9;11(4):969. doi: 10.3390/nano11040969 (PMC8070439; doi:10.3390/nano11040969)
Supplement: Supplementary file 1 [file nanomaterials-11-00969-s001.pdf]

Supplementary Material

Teeth of both species were placed in a mould, covered in Spurrs resin and put in an oven at 60 °C for 12 hours to allow the resin to set. The teeth were then ground in the longitudinal and transverse sections and the exposed areas were polished with 0.2 µm aluminium powder. The samples were sonicated in distilled water for 2 minutes and then etched with 10 % hydrochloric acid for 2 minutes and rinsed in distilled water for 5 minutes. They were carbon coated in an evaporation coater and viewed with a Tescan MIRA SEM at 5 kV.

Samples of *I. oxyrinchus* teeth were acquired from Hout Bay, South Africa. The *C. taurus* teeth samples had been shed naturally teeth and were kindly provided by Two Oceans Aquarium, Cape Town, South Africa. The teeth originate from one or more of the 5 adult females living in captivity (but born free) at the aquarium, weighing 80 kg to 170 kg. Teeth samples were stored at ambient conditions before specimen preparation and imaging.

This supplementary material contains further SEM figures taken during the study.

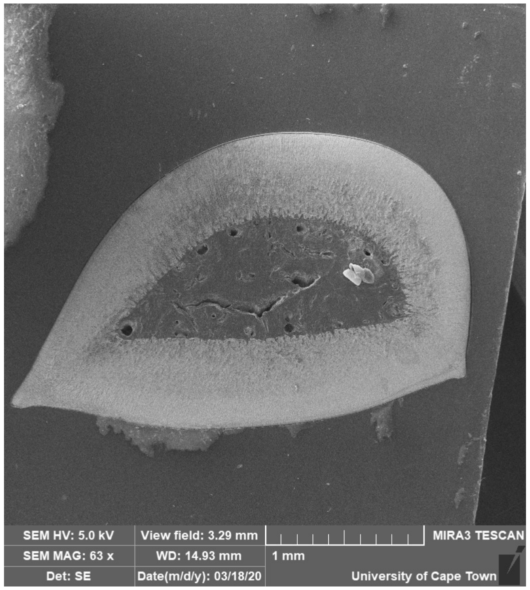

(a) overview

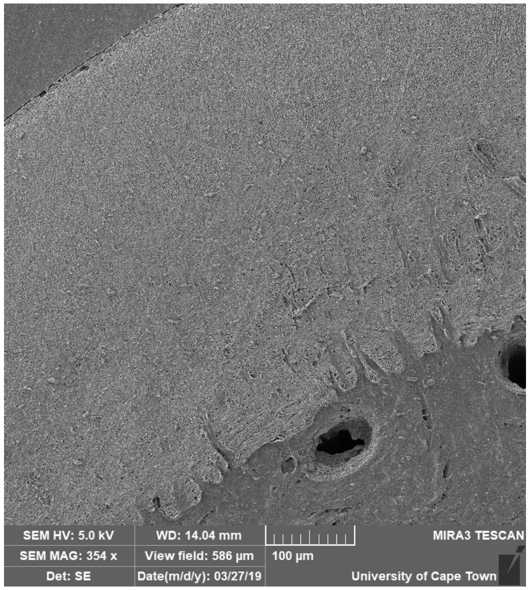

(b) enameloid and dentine

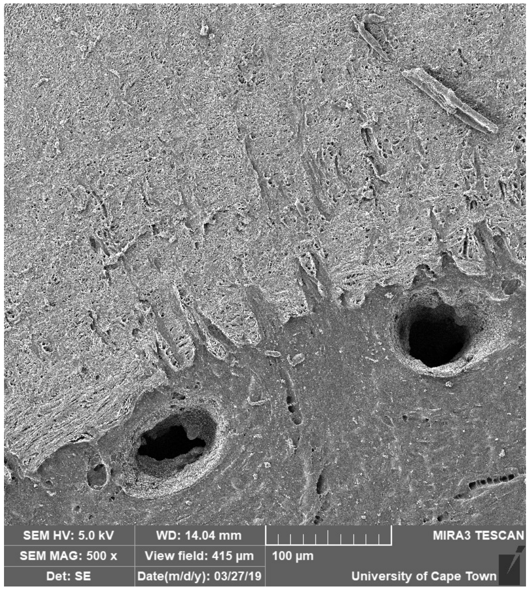

(c) inner enameloid close to dentine

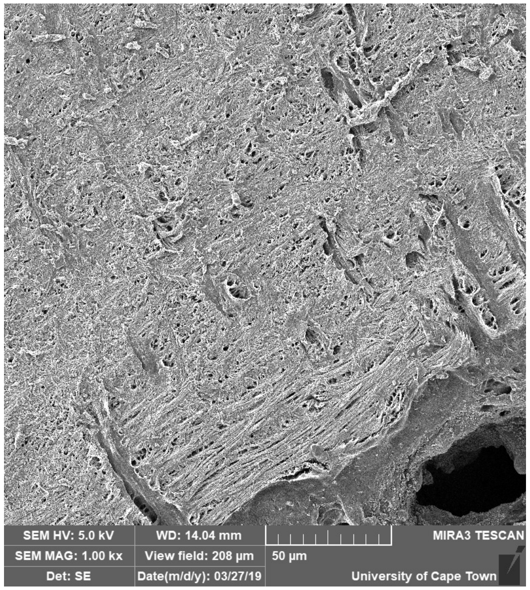

(d) inner enameloid close to dentine

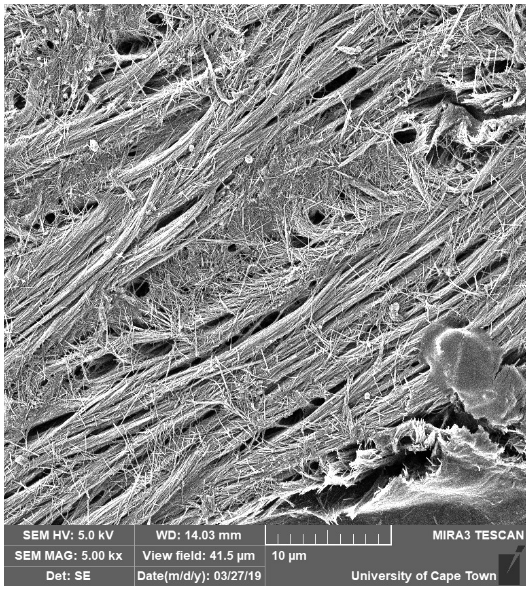

(e) zoom into inner enameloid

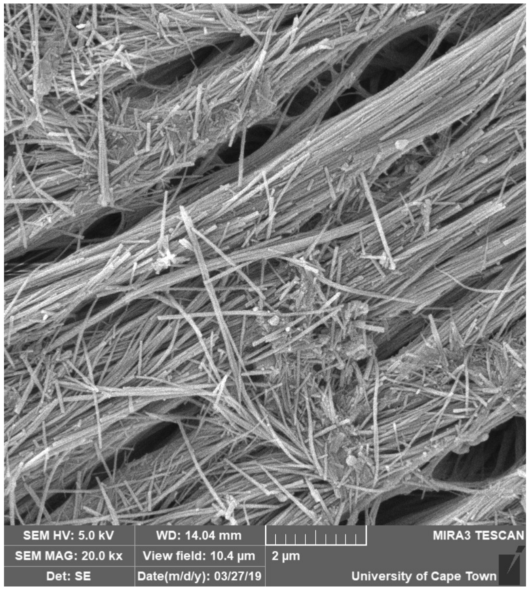

(f) zoom into inner enameloid

Figure S1: Enameloid of *I. oxyrinchus* in transverse section.

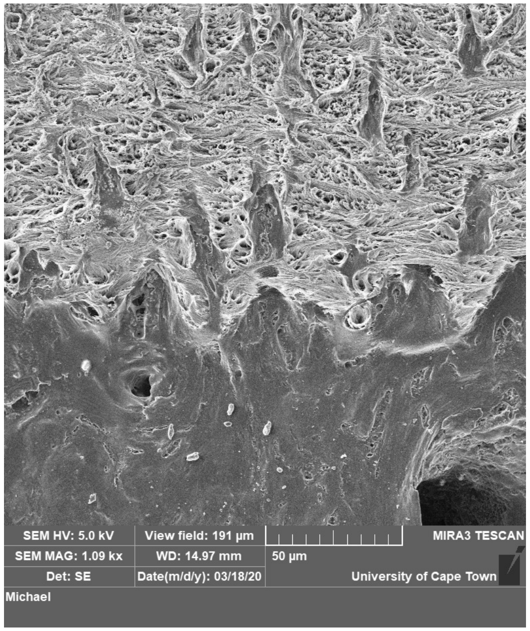

(a) enameloid-dentine junction

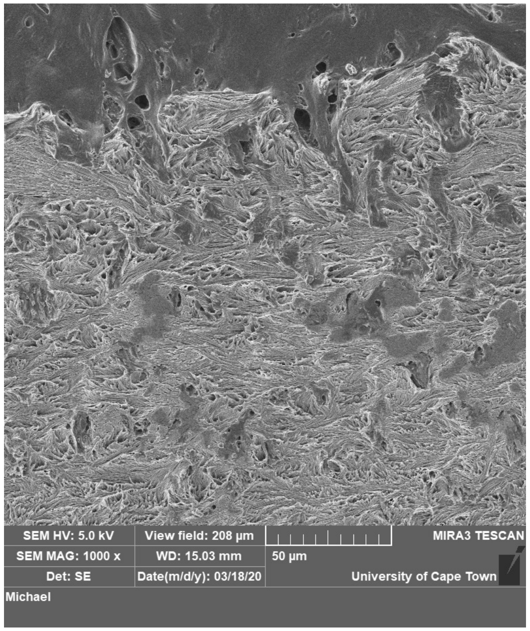

(b) enameloid-dentine junction

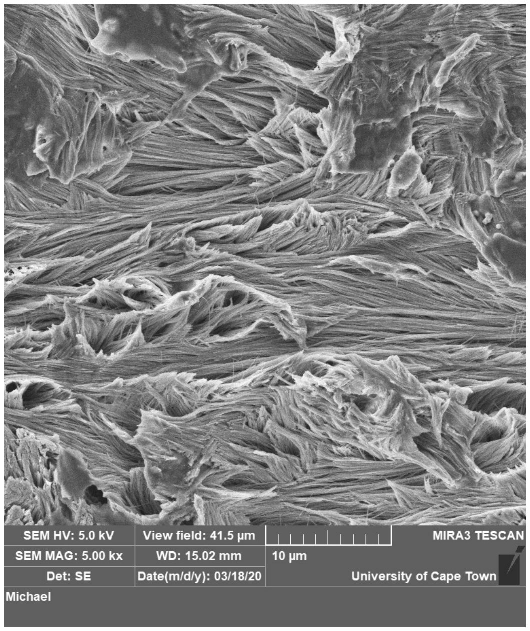

(c) TBE at enameloid-dentine junction (zoom into S2(b))

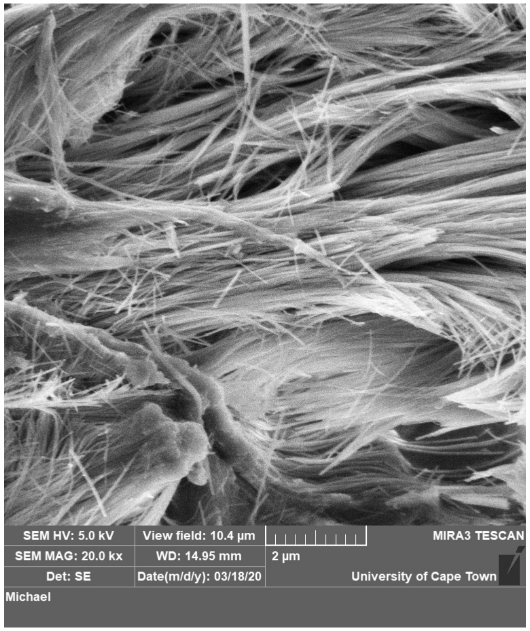

(d) TBE at enameloid-dentine junction

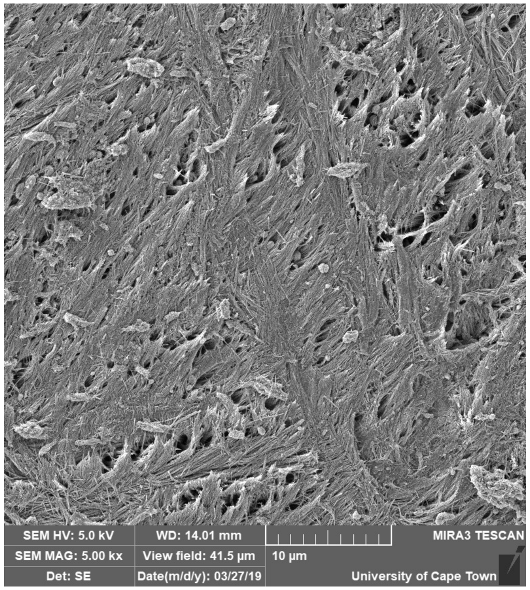

(e) middle region of inner enameloid

Figure S2: Enameloid of *I. oxyrinchus* in transverse section.

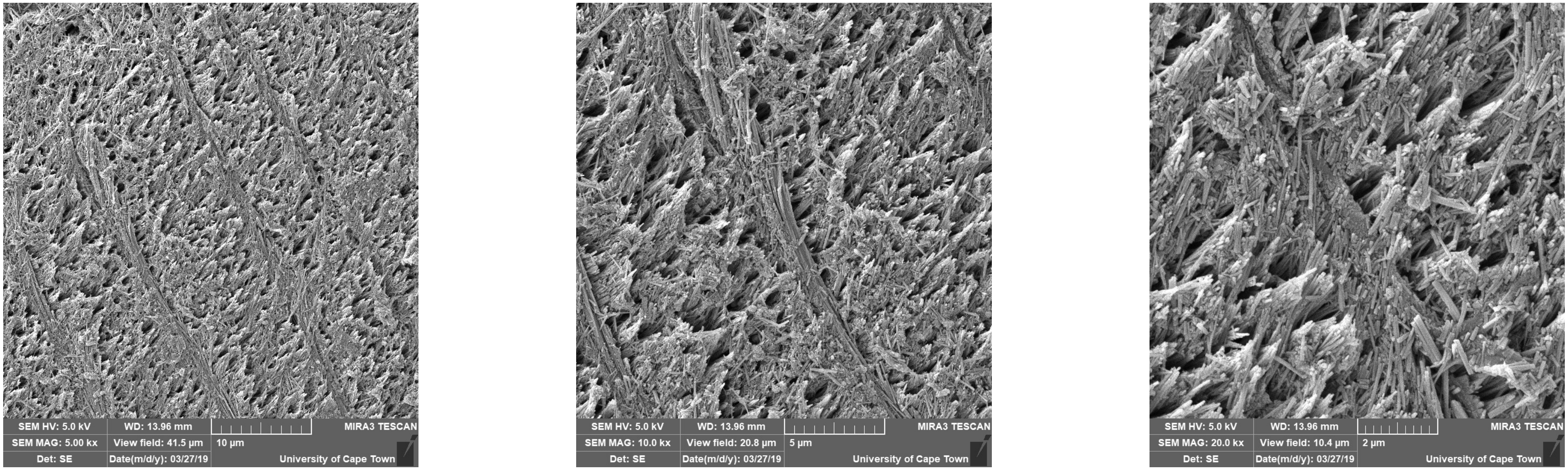

(a) outer enameloid and radial elements close to surface

(b) radial elements (zoom into S3(e))

(c) radial elements (zoom into S3(f))

Figure S3: Enameloid of *I. oxyrinchus* in transversal section.

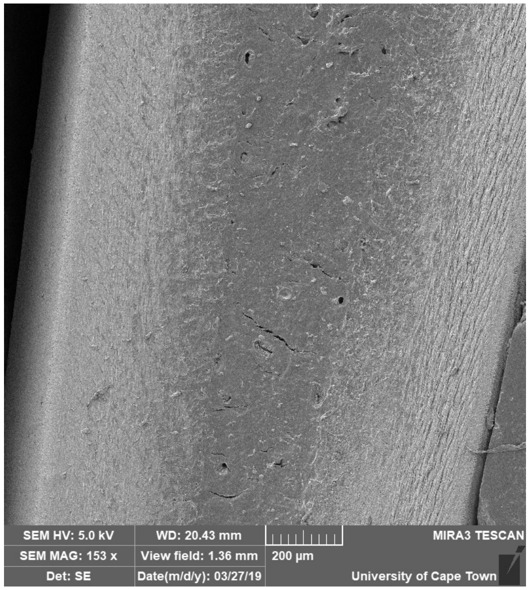

(a) overview of tooth cross section

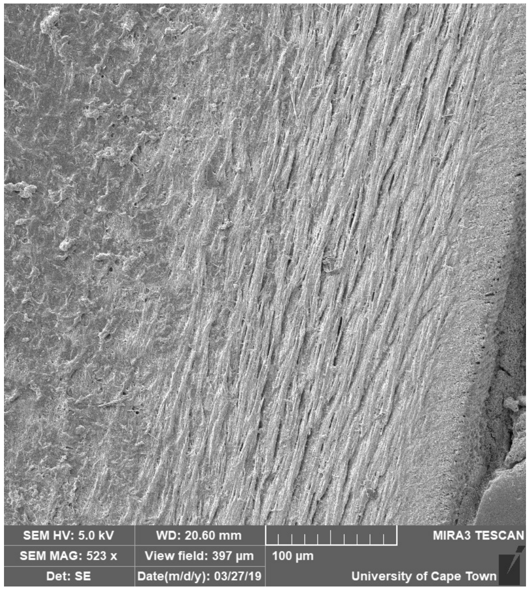

(b) layered structure of enameloid

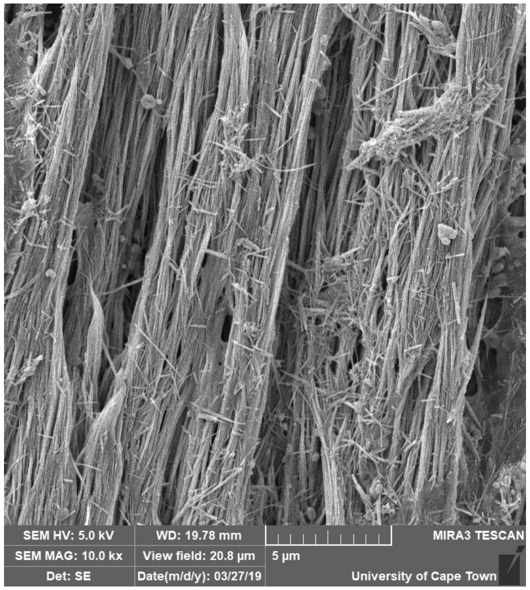

(c) inner enameloid in center region

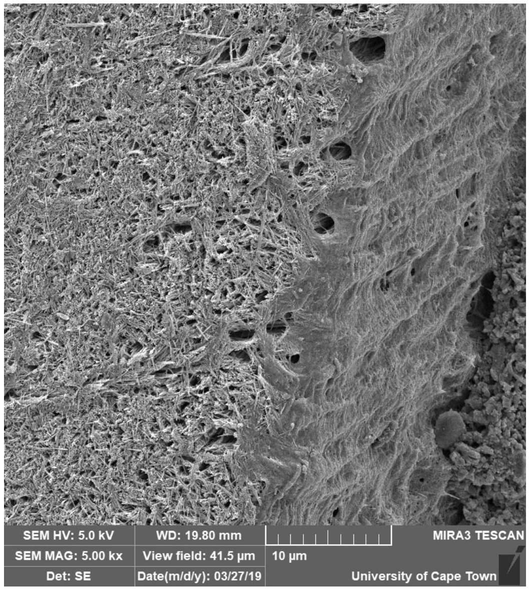

(d) edge of enameloid in upper tooth region

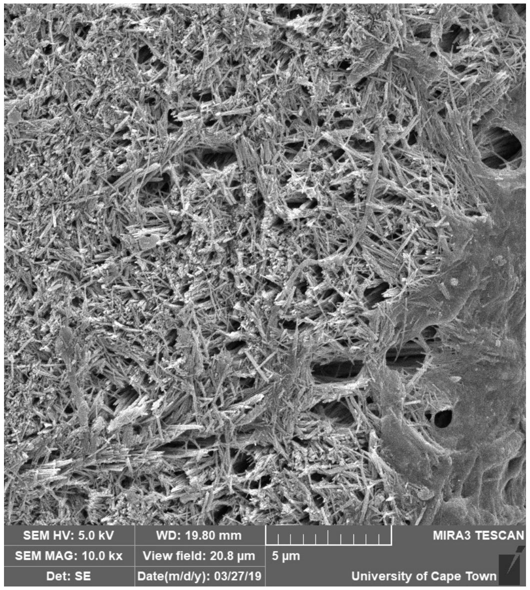

(e) edge of enameloid in upper tooth region

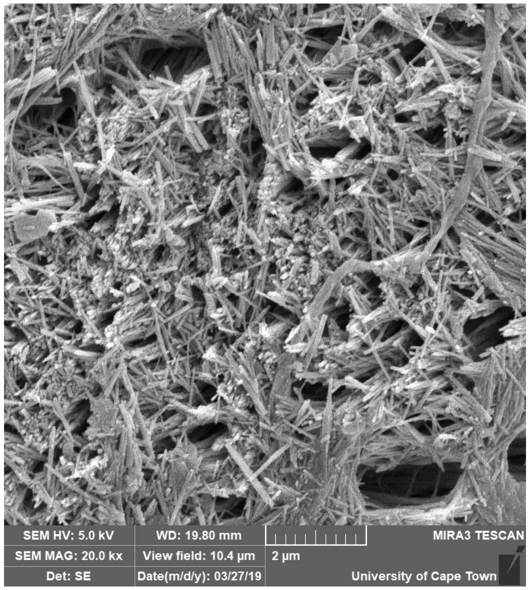

(f) edge of enameloid in upper tooth region

Figure S4: Enameloid of *I. oxyrinchus* in longitudinal section close to tip of tooth.

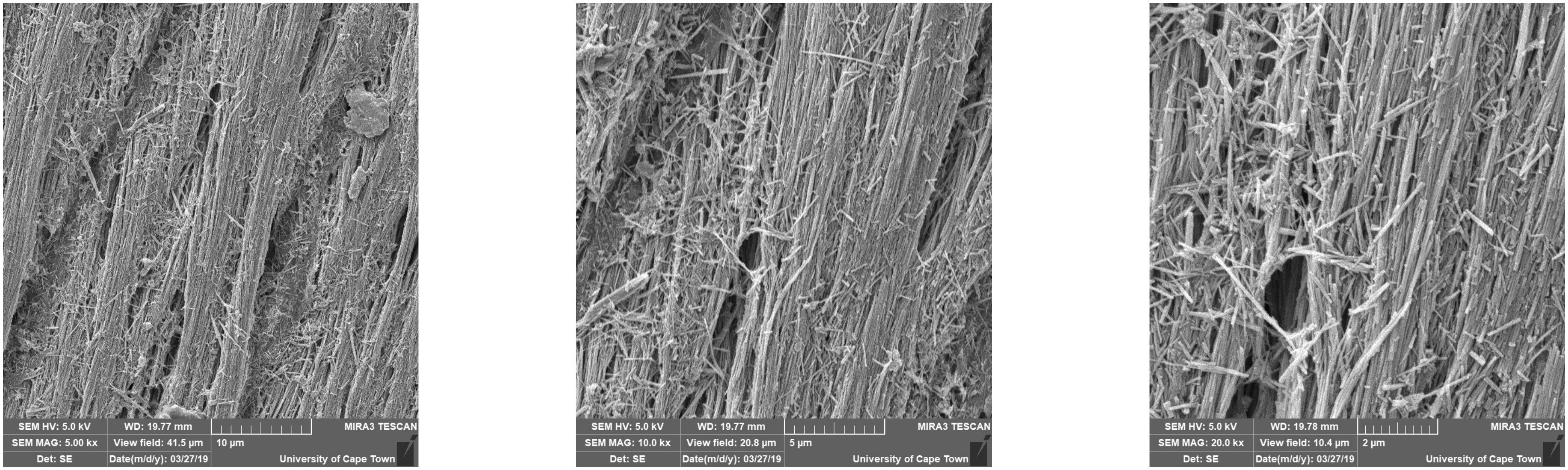

(a) parallel bundles in inner enameloid

(b) inner enameloid (zoom into S5(a))

(c) inner enameloid (zoom into S5(b))

Figure S5: Enameloid of *I. oxyrinchus* in longitudinal section close to tip of tooth.

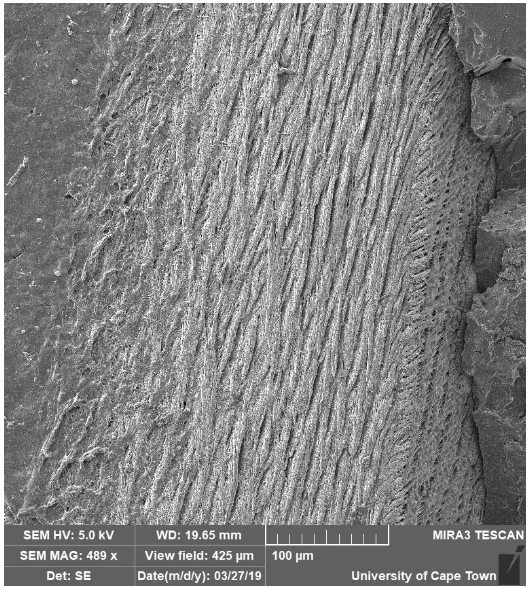

(a) layered structure of enameloid

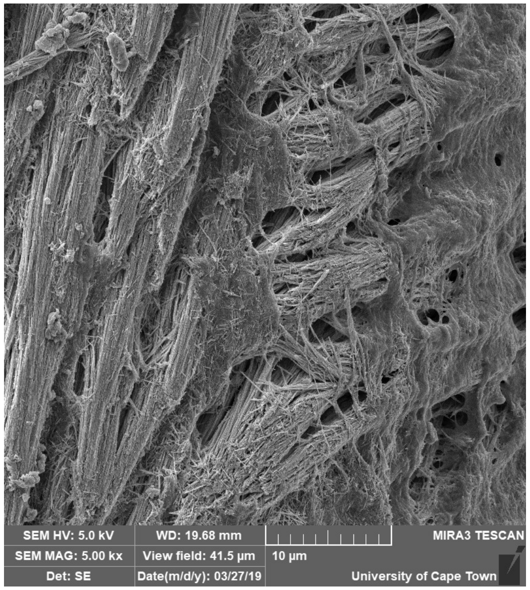

(b) edge of enameloid in lower tooth region

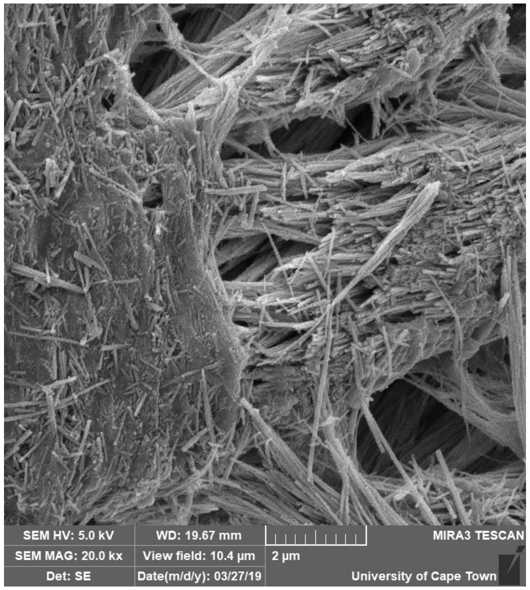

(c) edge of enameloid in lower tooth region

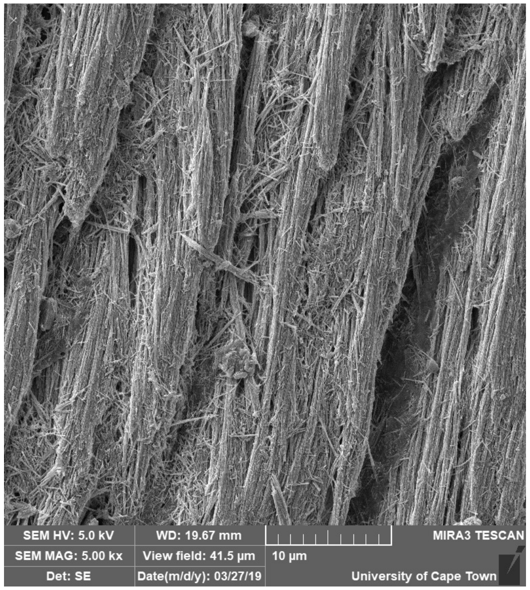

(d) parallel bundles in inner enameloid

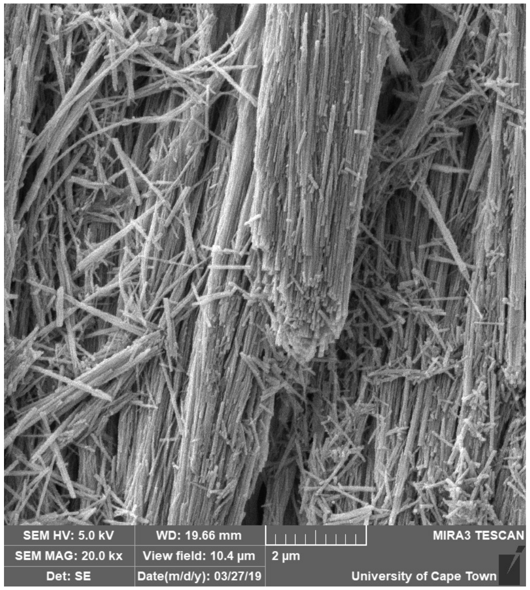

(e) inner enameloid (zoom into S6(d))

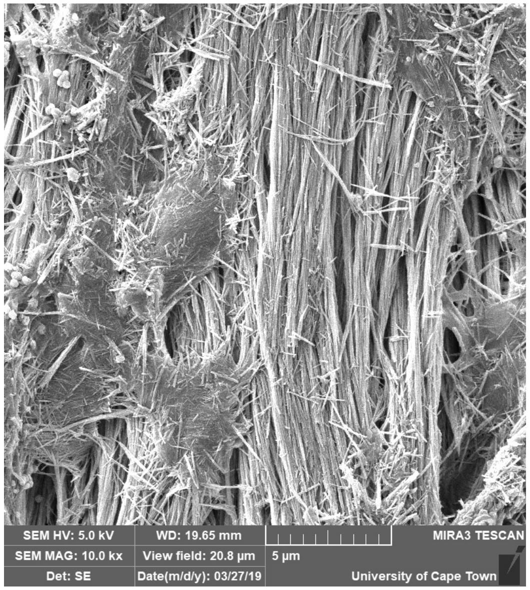

(f) inner enameloid close to dentine

Figure S6: Enameloid of *I. oxyrinchus* in longitudinal section in lower part of the tooth.

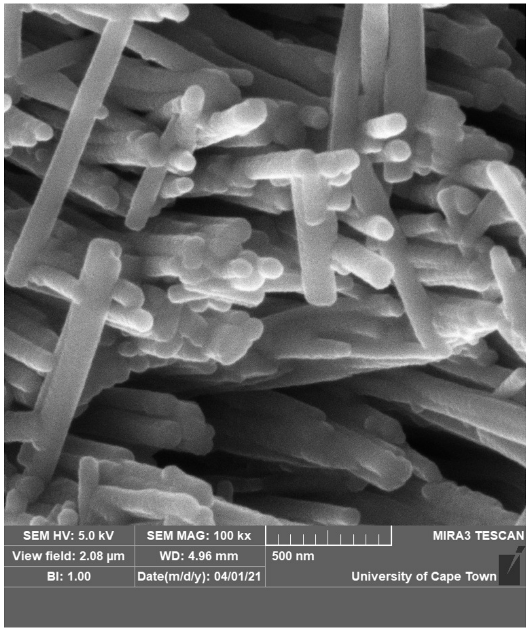

(a) crystallites on polished surface

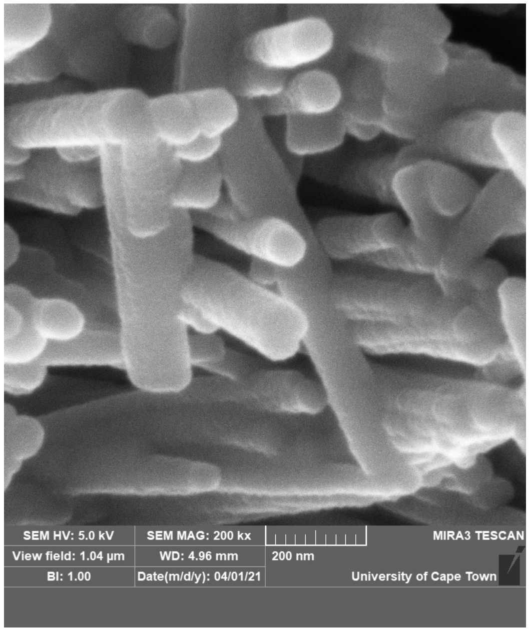

(b) crystallites on polished surface

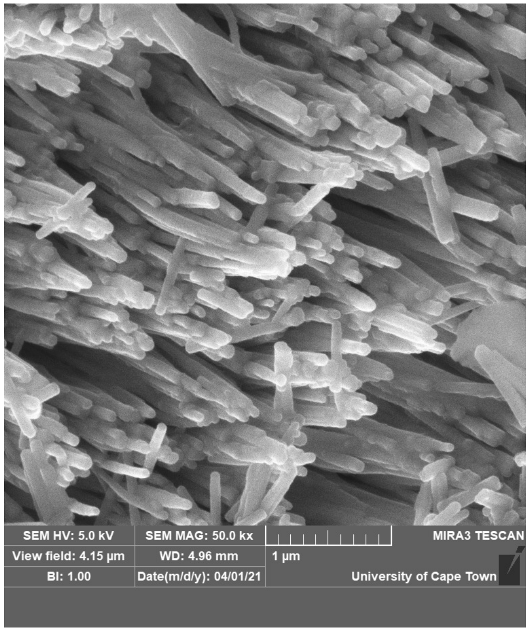

(c) aligned crystallites within a bundle (PBE in longitudinal section)

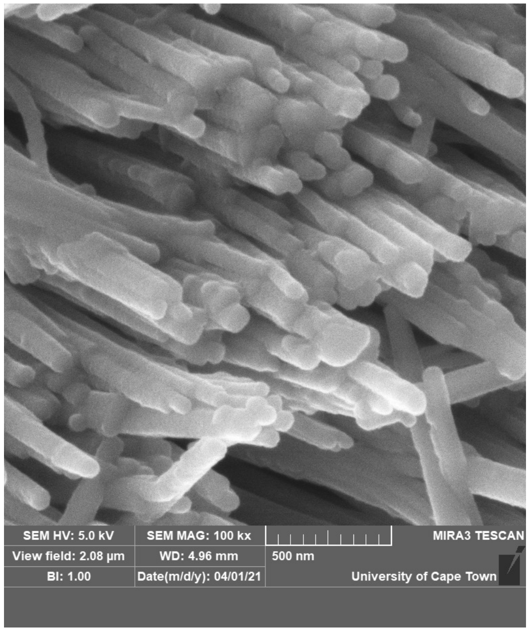

(d) aligned crystallites within a bundle (PBE in longitudinal section)

Figure S7: Crystallites in *I. oxyrinchus* enameloid.

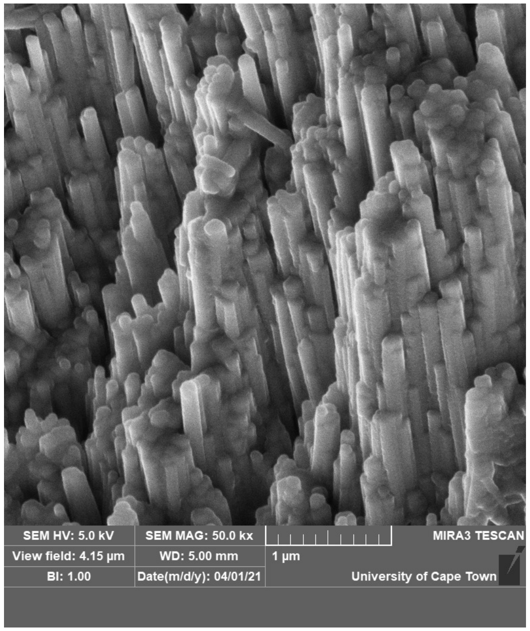

(a) aligned crystallites within a bundle (transversal section)

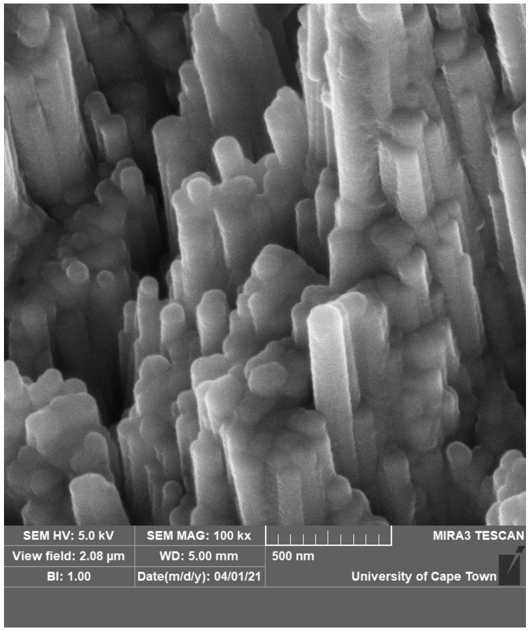

(b) aligned crystallites within a bundle (transversal section)

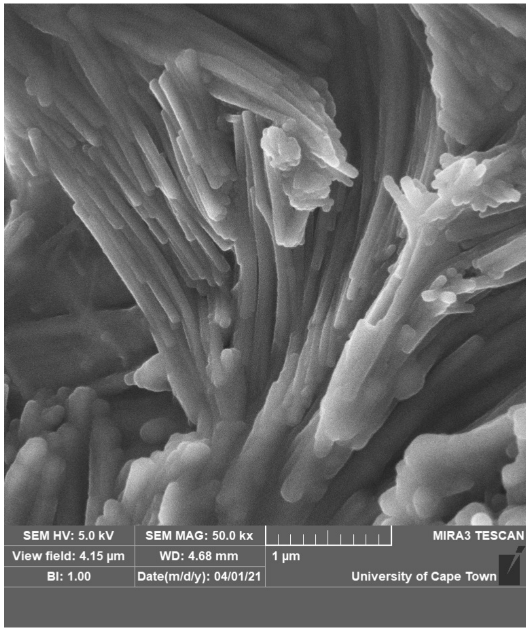

(c) crystallites in TBE (transversal section)

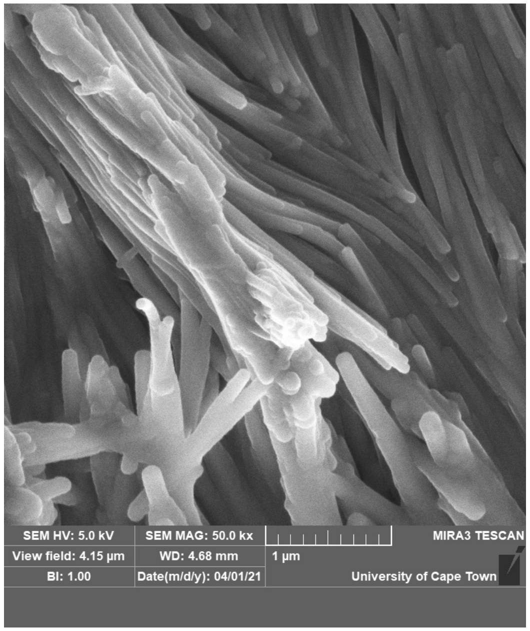

(d) crystallites in TBE (transversal section)

Figure S8: Crystallites in *I. oxyrinchus* enameloid.

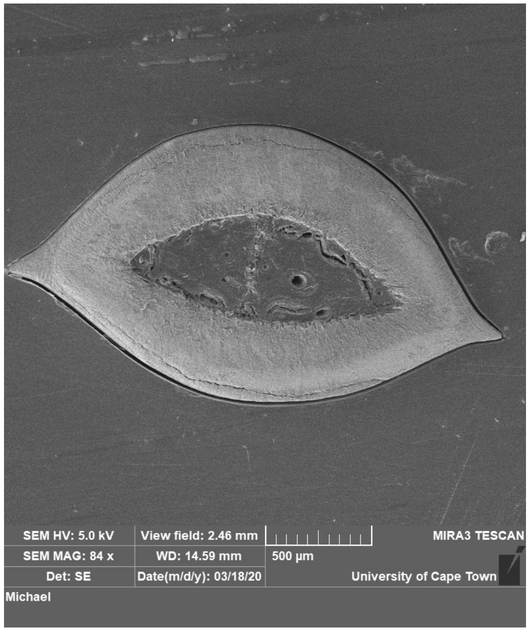

(a) overview

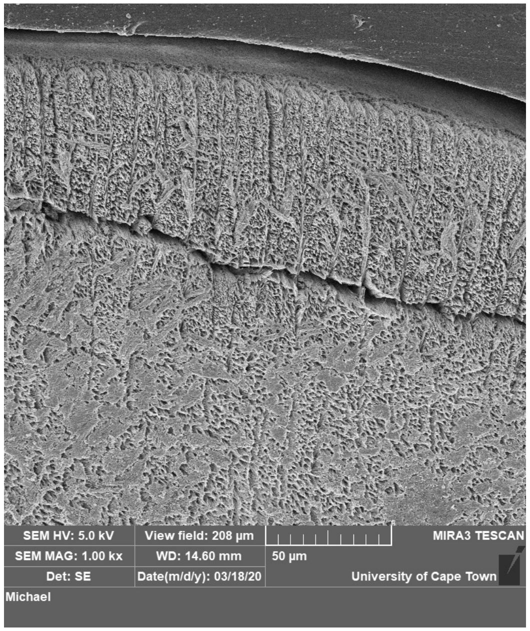

(b) edge of the tooth

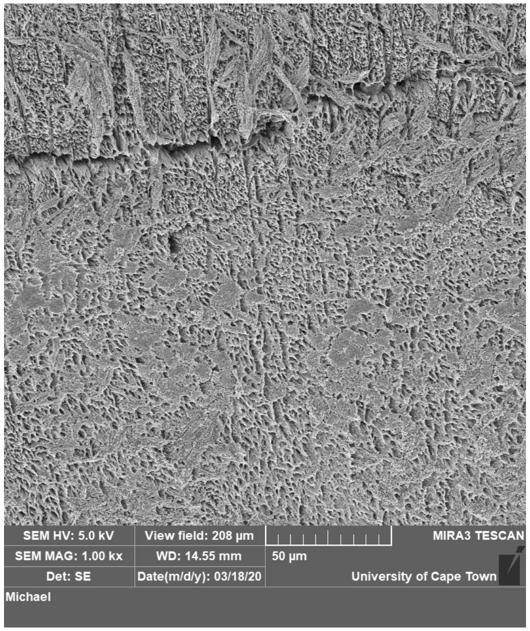

(c) enameloid close to the edge

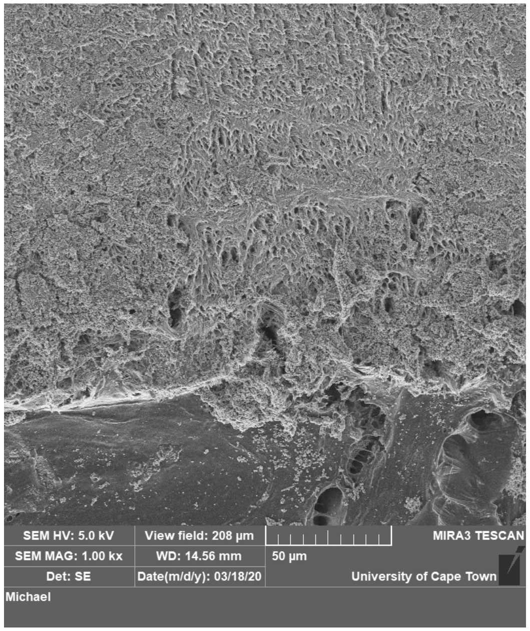

(d) inner enameloid close to dentine

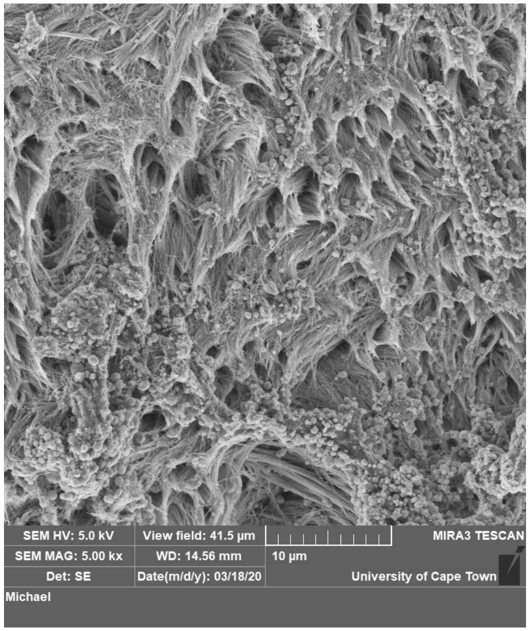

(e) inner enameloid close to dentine

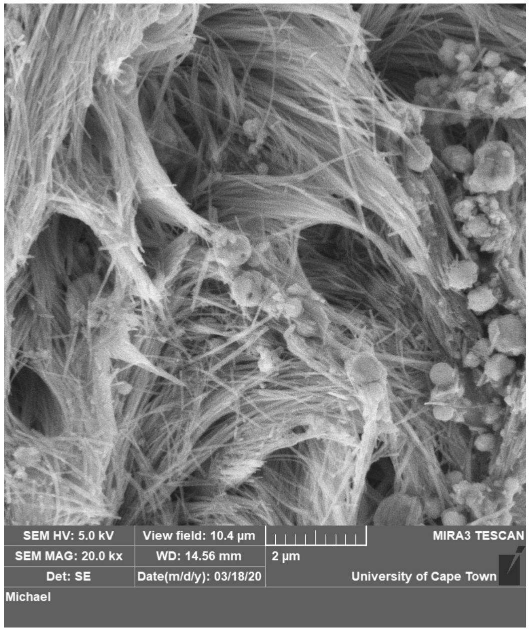

(f) inner enameloid close to dentine

Figure S9: Enameloid of *C. taurus* in transversal section.

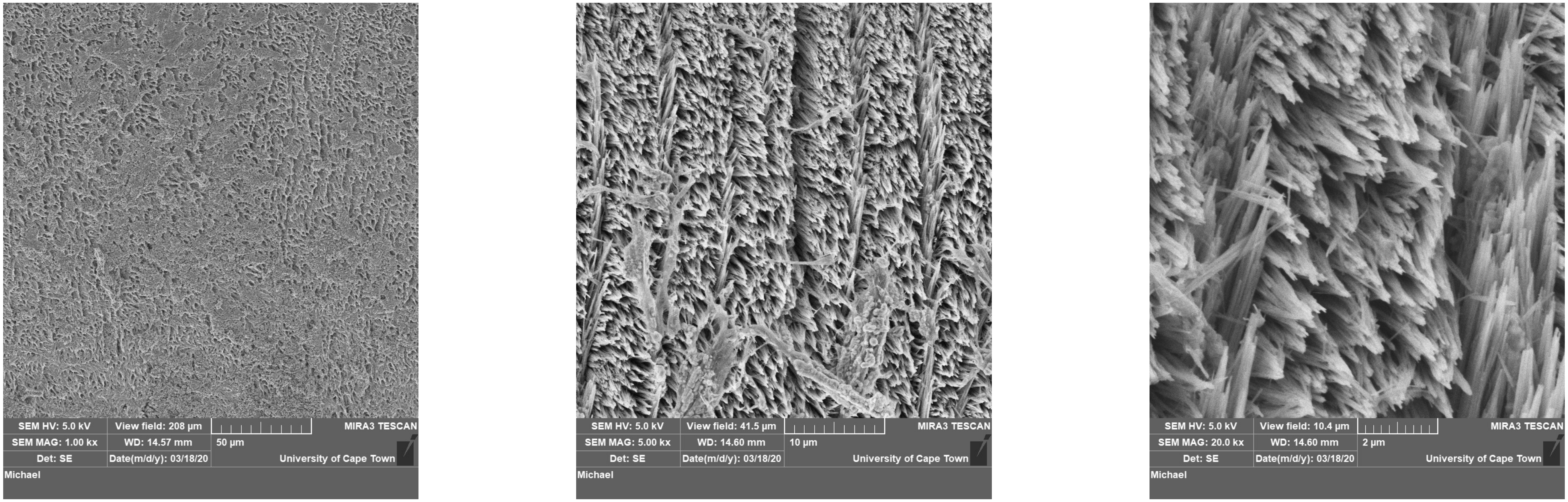

(a) middle of the enameloid cover

(b) radial elements close to edge

(c) radial elements close to edge

Figure S10: Enameloid of *C. taurus* in transversal section.

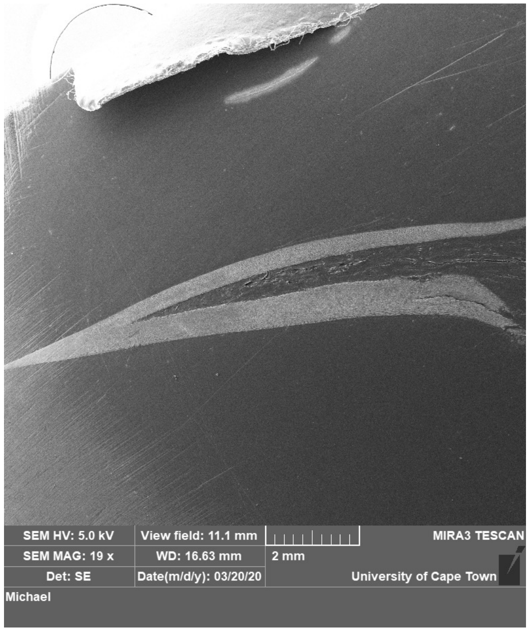

(a) overview

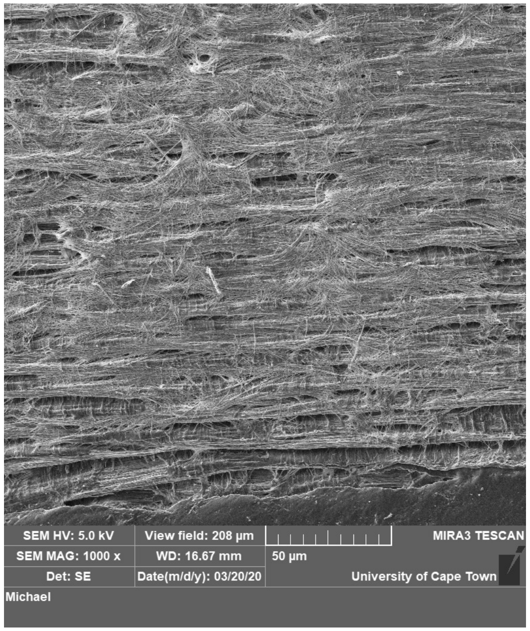

(b) edge of the tooth

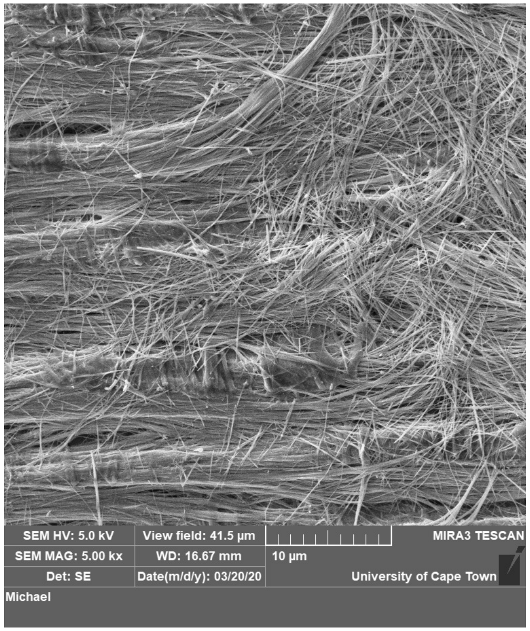

(c) zoom into (b)

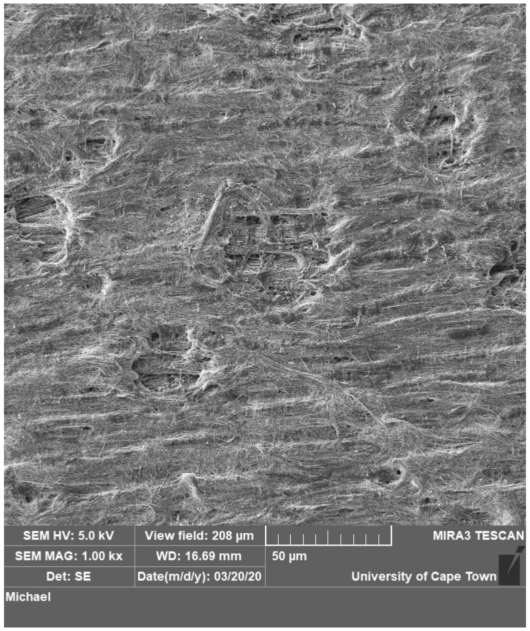

(d) parallel bundles in middle of enameloid

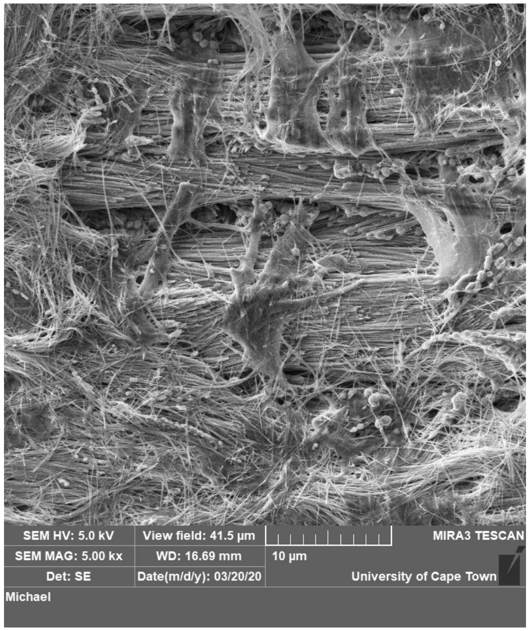

(e) inner enameloid (zoom into S11(d))

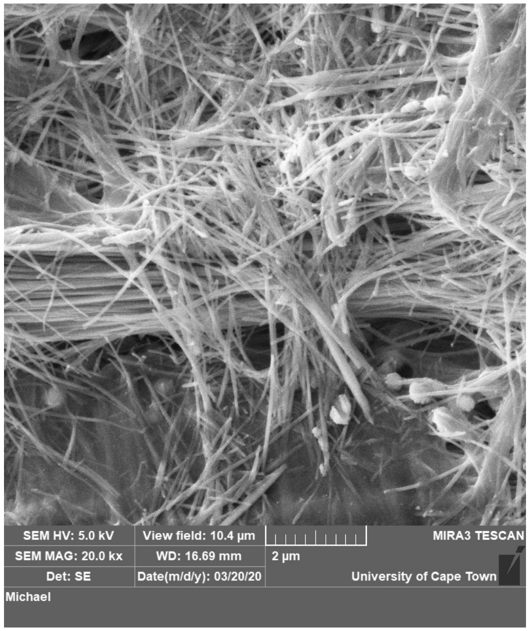

(f) inner enameloid (zoom into S11(e))

Figure S11: Enameloid of *C. taurus* in longitudinal section.

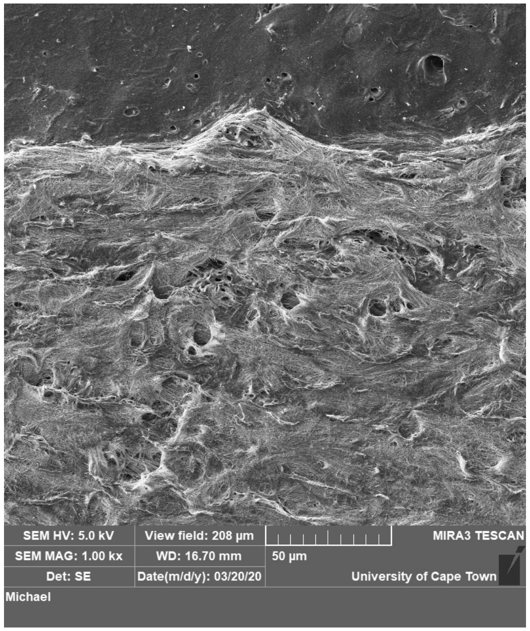

(a) enameloid dentine junction

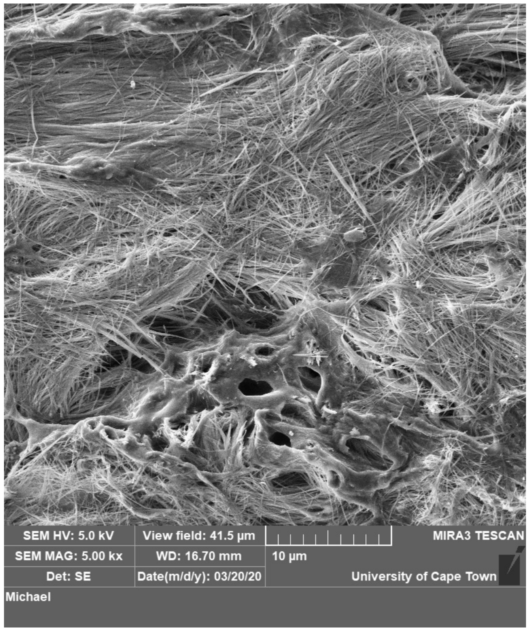

(b) inner enameloid close to dentine

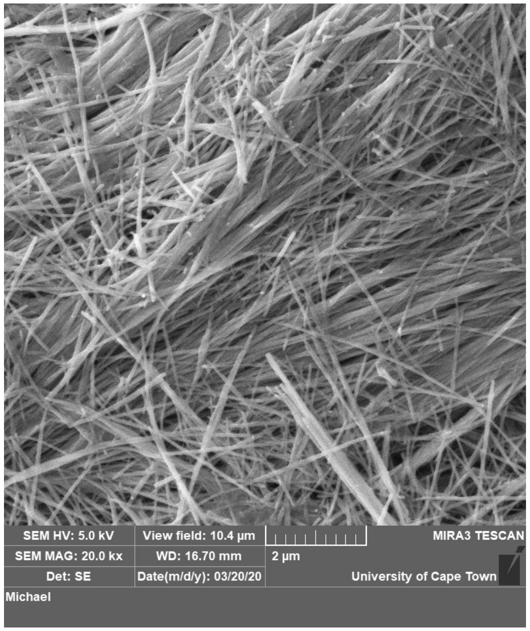

(c) inner enameloid (zoom into S12(b))

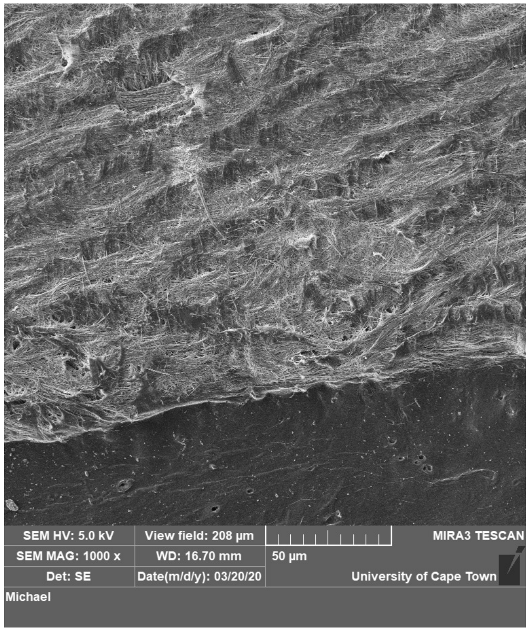

(d) enameloid dentine junction

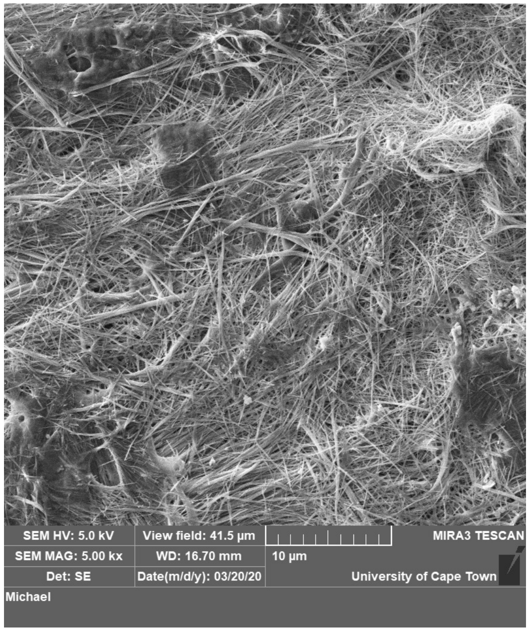

(e) inner enameloid close to dentine

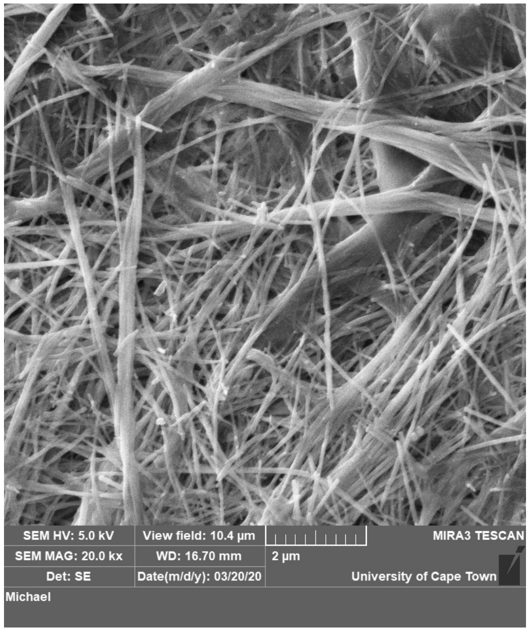

(f) inner enameloid (zoom into S12(e))

Figure S12: Enameloid of *C. taurus* in longitudinal section in two areas of the tooth.

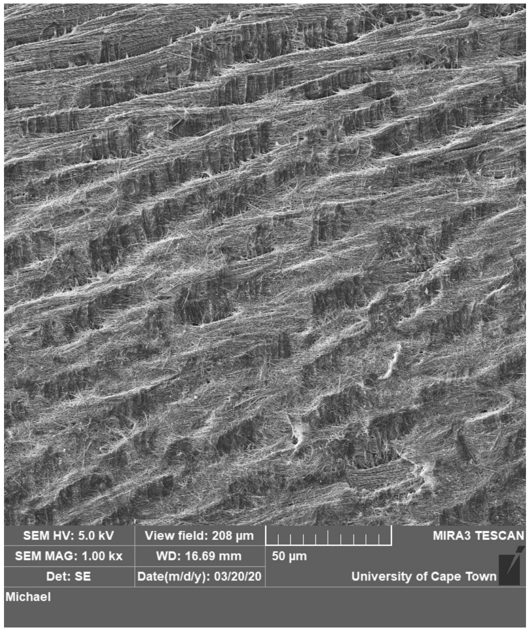

(a) parallel bundles close to tooth edge

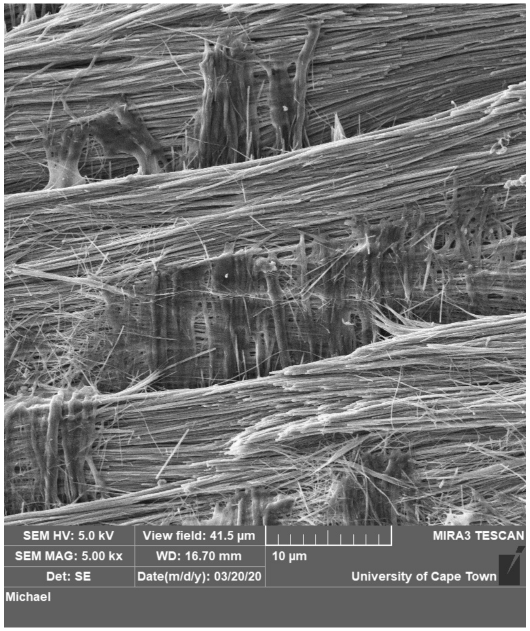

(b) parallel bundles close to tooth edge

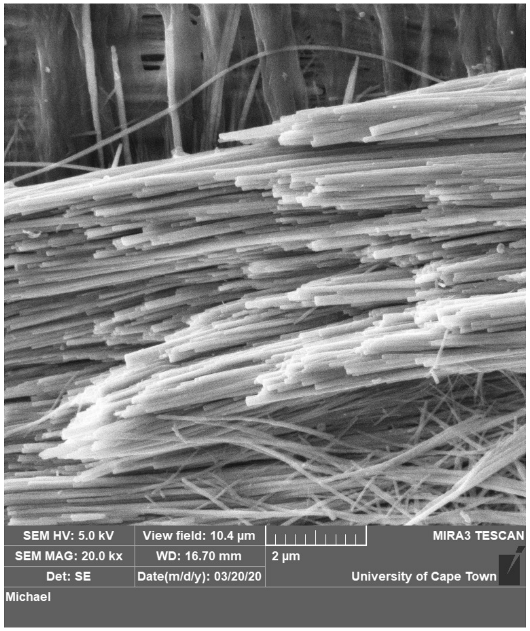

(c) zoom into individual bundle in S13(b)

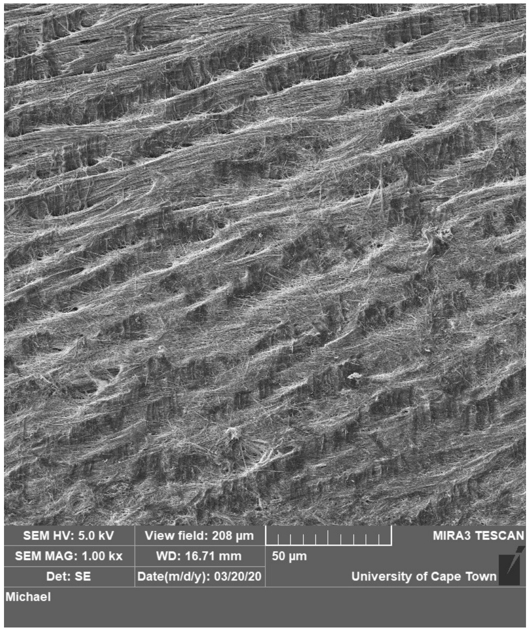

(d) parallel bundles further inside the enameloid cover

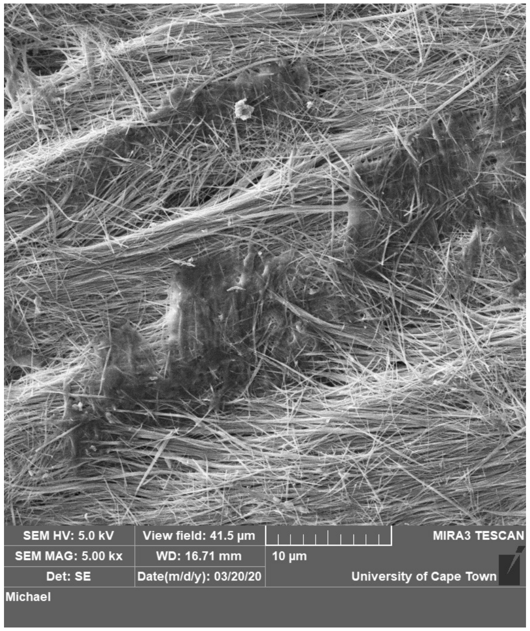

(e) parallel bundles further inside the enameloid cover

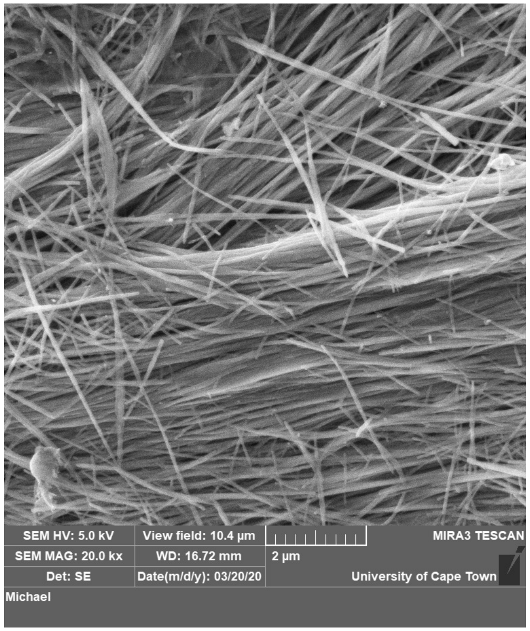

(f) zoom into individual bundle in S13(e)

Figure S13: Enameloid of *C. taurus* in longitudinal section.

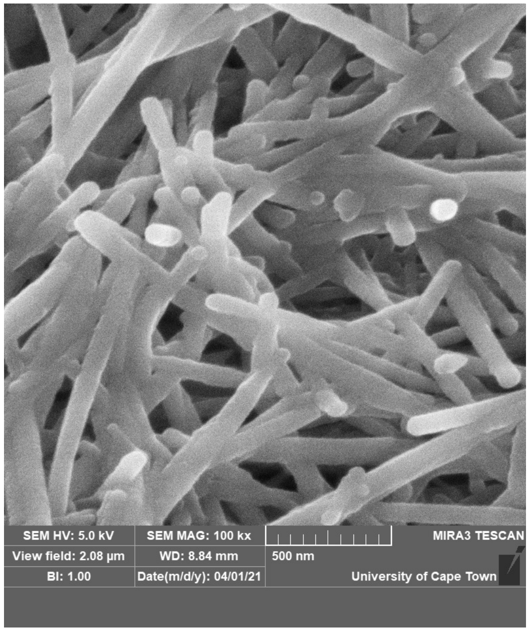

(a) crystallites on polished surface

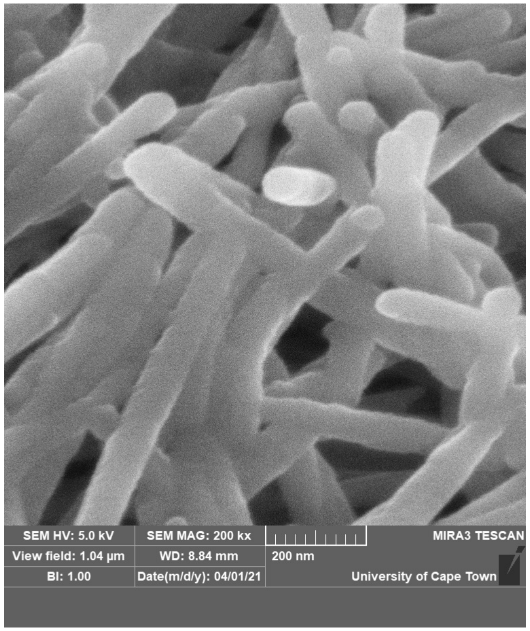

(b) crystallites on polished surface

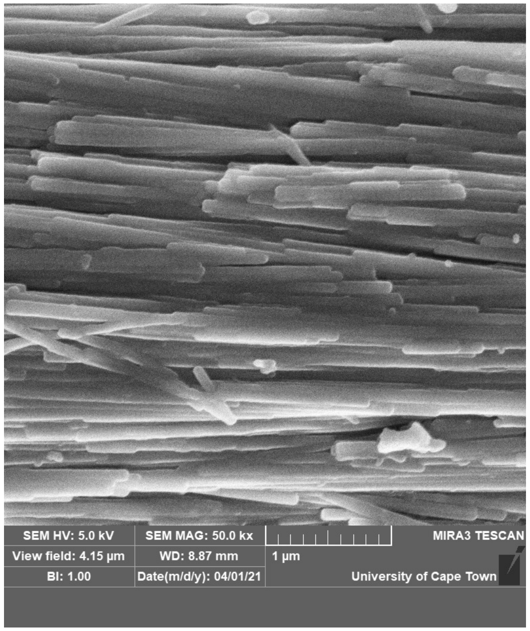

(c) aligned crystallites within a bundle (PBE in longitudinal section)

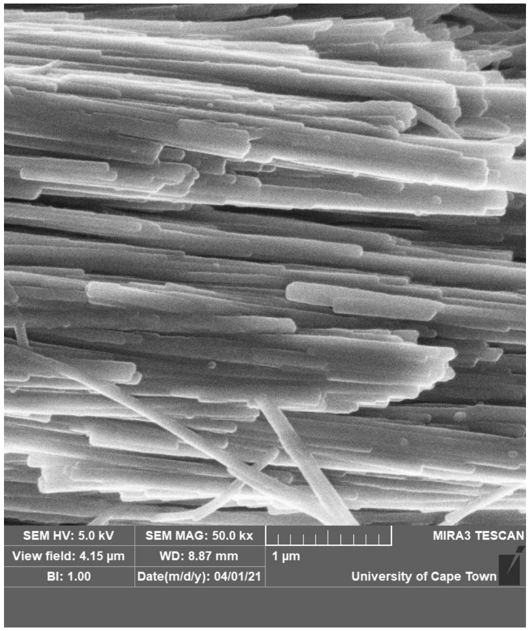

(d) aligned crystallites within a bundle (PBE in longitudinal section)

Figure S14: Crystallites in *C. taurus* enameloid.

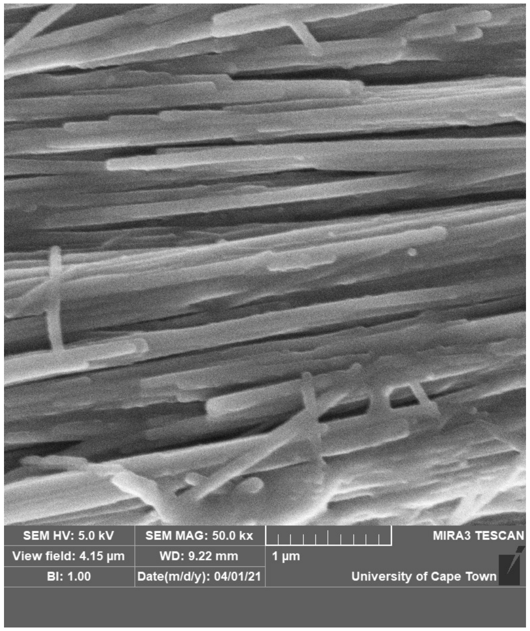

(a) aligned crystallites within a bundle (transversal section)

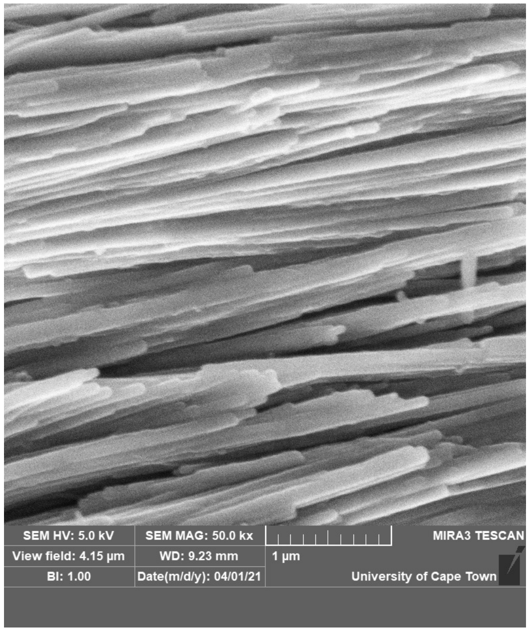

(b) aligned crystallites within a bundle (transversal section)

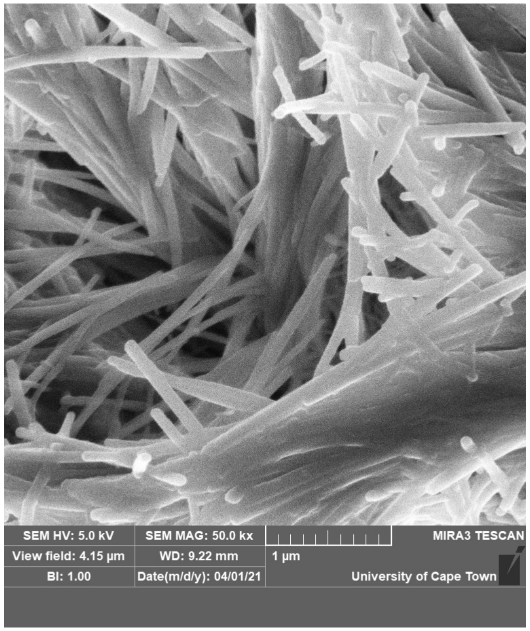

(c) crystallites in TBE (transversal section)

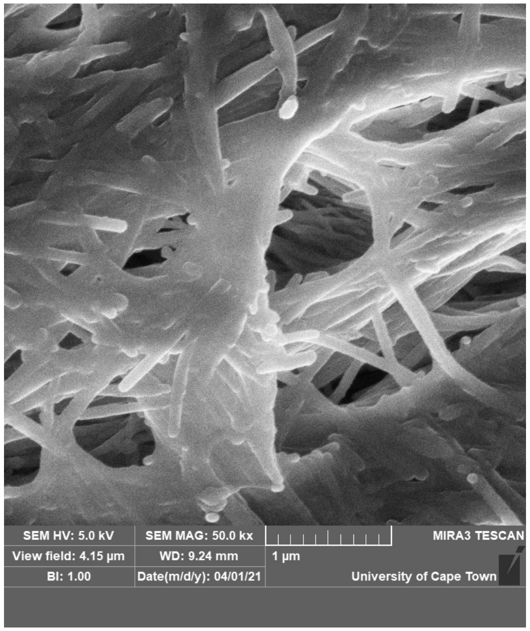

(d) crystallites in TBE (transversal section)

Figure S15: Crystallites in *C. taurus* enameloid.
